# Anti-enterovirus 71 activities of *Melissa officinalis* extract and its biologically active constituent rosmarinic acid

**DOI:** 10.1038/s41598-017-12388-2

**Published:** 2017-09-25

**Authors:** Sin-Guang Chen, Yann-Lii Leu, Mei-Ling Cheng, Siew Chin Ting, Ching-Chuan Liu, Shulhn-Der Wang, Cheng-Hung Yang, Cheng-Yu Hung, Hiroaki Sakurai, Kuan-Hsing Chen, Hung-Yao Ho

**Affiliations:** 1 Graduate Institute of Biomedical Science, Chang Gung University, Guishan, Taoyuan, Taiwan; 2 Graduate Institute of Natural Products, College of Medicine, Chang Gung University, Taoyuan, Taiwan; 3 Center for Traditional Chinese Medicine, Chang Gung Memorial Hospital at Linkou, Guishan, Taoyuan, Taiwan; 4 Chinese Herbal Medicine Research Team, Healthy Aging Research Center, Chang Gung University, Taoyuan, Taiwan; 5 Department of Biomedical Sciences, College of Medicine, Chang Gung University, Guishan, Taoyuan, Taiwan; 6 Healthy Aging Research Center, Chang Gung University, Guishan, Taoyuan, Taiwan; 7 Metabolomics Core Laboratory, Chang Gung University, Guishan, Taoyuan, Taiwan; 8 Clinical Phenome Center, Chang Gung Memorial Hospital at Linkou, Guishan, Taoyuan, Taiwan; 9 Department of Medical Biotechnology and Laboratory Science, College of Medicine, Chang Gung University, Taoyuan, Taiwan; 10 Department of Pediatrics, National Cheng Kung University Hospital, College of Medicine, National Cheng Kung University, Tainan, Taiwan; 11 Center of Infectious Disease and Signaling Research, National Cheng Kung University, Tainan, Taiwan; 12 School of Post-Baccalaureate Chinese Medicine, College of Chinese Medicine, China Medical University, Taichung, Taiwan; 13 Department of Cancer Cell Biology, Graduate School of Medicine and Pharmaceutical Sciences, University of Toyama, Toyama, Japan; 14 Kidney Research Center, Chang Gung Memorial Hospital, Chang Gung University, School of Medicine, Taoyuan, Taiwan

## Abstract

Enterovirus 71 (EV71) infection is endemic in the Asia-Pacific region. No specific antiviral drug has been available to treat EV71 infection. *Melissa officinalis* (MO) is a medicinal plant with long history of usage in the European and Middle East. We investigated whether an aqueous solution of concentrated methanolic extract (MOM) possesses antiviral activity. MOM inhibited plaque formation, cytopathic effect, and viral protein synthesis in EV71-infected cells. Using spectral techniques, we identified rosmarinic acid (RA) as a biologically active constituent of MOM. RA reduced viral attachment and entry; cleavage of eukaryotic translation initiation factor 4 G (eIF4G); reactive oxygen species (ROS) generation; and translocation of heterogeneous nuclear ribonucleoprotein A1 (hnRNP A1) from nucleus to cytoplasm. It alleviated EV71-induced hyperphosphorylation of p38 kinase and EPS15. RA is likely to suppress ROS-mediated p38 kinase activation, and such downstream molecular events as hnRNP A1 translocation and EPS15-regulated membrane trafficking in EV71-infected cells. These findings suggest that MO and its constituent RA possess anti-EV71 activities, and may serve as a candidate drug for therapeutic and prophylactic uses against EV71 infection.

## Introduction

Hand, foot, and mouth disease (HFMD) is a prevalent infectious childhood disease caused by several viral strains belonging to the genus *Enterovirus* within the family *Picornaviridae*. Enterovirus 71 (EV71) is one of the major pathogens causing HFMD in infants and children aged under 5^1,2^. Patients with HFMD suffered from fever and vesicular exanthemas on hands, feet, buttocks and mouth^3^. A small proportion of EV71-infected patients develop severe central nervous system (CNS) infection, which can cause neurological cardiopulmonary failure, systemic complication, and even fatality^4,5^. Even those who survive severe infection may have long-term motor and cognitive deficits^6–8^. EV71 was first isolated from patients with CNS disease in California, USA, during 1969 to 1972^9^, and caused several sporadic cases around the world in the 1970s and 1980s^10^. In recent two decades, severe EV71 outbreaks have affected more than millions of children, and claimed hundreds of lives in the Asia-Pacific region^2,11–15^. There has not been specific treatment for enteroviral CNS infection, and supportive therapies have been used to manage EV71-induced pulmonary heart failure^16^. Development of novel therapeutics is needed to improve the clinical outcome of infection.

EV71 is a positive single-stranded RNA virus and its genome is approximately 7,400 nucleotides long. The RNA genome is enclosed in an icosahedral capsid shell. During viral infection, EV71 attaches to cellular receptors and enter host cells through endocytosis^17^. Engagement of virus with scavenger receptor B2 (SCARB2) receptor results in release of viral RNA^18^. Viral RNA contains a highly structured type I internal ribosomal entry site (IRES) within 5′ untranslated region (UTR). The IRES interacts with host proteins, namely IRES *trans*-acting factors (ITAFs), to recruit ribosome and initiate translation^19,20^. Heterogeneous nuclear ribonucleoprotein (hnRNP) A1, an RNA binding protein, acts as an ITAF to enhance EV71 IRES-mediated translation. Translocation of hnRNP A1 from nucleus to cytoplasm is required for viral translation and replication during EV71 infection^21,22^. Viral RNA is translated to a single precursor polyprotein that is hydrolyzed by viral proteases, 2A^pr^° and 3C^pr^°, to produce mature viral structural proteins (VP1, VP2, VP3 and VP4) and nonstructural proteins (2A, 2B, 2C, 3A, 3B, 3C and 3D)^20^. EV71 utilizes the translation apparatus of host cells to produce viral proteins^23^, and switches the translation of capped mRNA to viral RNA. Viral proteases, 2A^pr^° and 3C^pr^°, cleave eukaryotic initiation factor 4 G (eIF4G), eukaryotic initiation factor 4A (eIF4A) and poly(A)-binding protein (PABP), which are required for cap-dependent translation^24^. EV71 infection induces expression of miR-141 which targets eukaryotic initiation factor 4 G (eIF4G)^25^.

Several lines of evidence support the notion that the redox status of host affects viral pathogenesis^26^. We have previously shown that EV71 infection induces oxidative stress, which in turn promotes viral replication^27,28^. Treatment with antioxidants, such as N-acetylcysteine (NAC) or mito-Tempo, suppresses EV71 replication^27–29^. Natural antioxidants, epigallocatechin gallate (EGCG) and gallocatechin gallate (GCG), in green tea have antiviral activity, which correlates well with their antioxidant capacity^30^. It is probable that the antiviral activities of natural products may be partly attributed to their antioxidative activities.

Herbal plants are potential sources for development of antiviral drugs^31^. *Melissa officinalis* (MO), also known as lemon balm, is a perennial plant belonging to family Labiatae. In Southern Europe, Mediterranean region, Western Asia, and North Africa, fresh leaves of MO have been used to add flavor to dishes, herbal tea, vinegars, and oils for more than 2000 years. Dried or fresh leaves and stems of MO are used as medicine to treat inflammatory, gastrointestinal, mental, neuralgic, and rheumatic disorders^32^. MO displays an antiviral activity against herpes simplex virus type 1, herpes simplex virus type 2, human immunodeficiency virus type 1, and influenza virus^33–38^. Choi *et al*. showed that the aqueous extract of MO prevents the viability loss in EV71-infected Vero cells^39^. However, the mechanism underlying the antiviral activity of MO is currently unknown.

In present study, we show that methanolic extract of MO (MOM) inhibits cytopathic effect, plaque formation, and viral protein synthesis in EV71-infected cells. Further characterization and identification reveals rosmarinic acid (RA) as an active antienteroviral constituent of MOM. Mechanistically, RA inhibits EV71-induced ROS accumulation, translocation of hnRNP A1, cleavage of eIF4G, and p38-mediated phosphorylation of epidermal growth factor receptor substrate 15 (EPS15). Our results suggest that RA acts on multiple pathways to exert its antiviral effect.

## Results

### Anti-EV71 activity of methanolic extract of MO (MOM)

**Figure 1:**
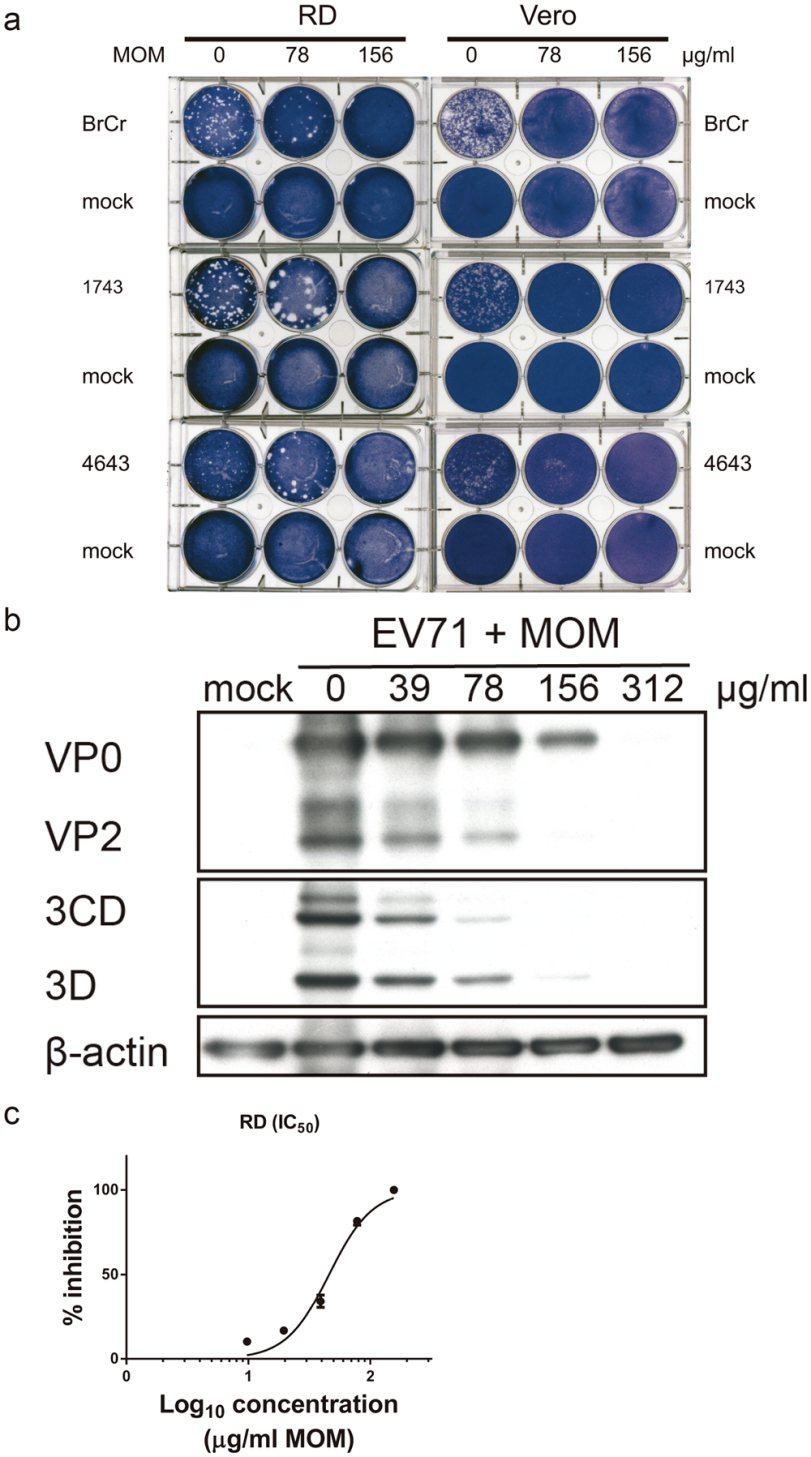
MOM inhibits EV71 infection. (**a**) RD and Vero cells were mock- or infected with BrCr, 1743 and 4643 strains for 1 h, and were overlaid with 0.3% agarose in DMEM/2% FBS supplemented with 0, 78, or 156 μg/ml MOM. RD cells were incubated for 3 days, and Vero cells were cultured for 5 days. The plates were fixed with 10% formalin, and stained with 1% crystal violet solution. Representative plates are shown here. (**b**) RD cells were infected with BrCr at an m. o. i. of 0.05 in the presence of indicated concentrations of MOM for 16 h. Cellular protein was harvested, and was subject to western blotting with antibodies to 3D, VP2 and β-actin. The cropped images of the blots are shown. The full-length blots are presented in Supplementary Fig. S8. A representative experiment out of three is shown. (**c**) RD cells were infected with BrCr at an m. o. i. of 10 in the presence of 9.75, 19.5, 39, 78, or 156 μg/ml of MOM for 24 h. The percentage of inhibition was determined. The results are expressed as the mean ± SD of three independent experiments.

The syrup of MOM was diluted in water, and tested for its antiviral activity. Using the plaque reduction assay, we examined the anti-EV71 activity of MOM. RD or Vero cell monolayers were infected with 100 PFU/well or 200 PFU/well of EV71 strains, BrCr, 1743, or 4643, for 1 h, and were overlaid with semisolid media containing 0.3% agarose and 0, 78, or 156 μg/ml of MOM. MOM diminished plaque formed by these virus strains in RD and Vero cells at concentrations up to 156 μg/ml (Fig. 1a). At these concentrations, MOM was non-cytotoxic to RD cells (CC_50_ = 370.9 ± 1.07 μg/ml, Supplementary Fig. S1a) or Vero cells (CC_50_ = 555.4 ± 1.05 μg/ml, Supplementary Fig. S1b). Additionally, MOM inhibited virus-induced cytopathic effect (CPE) in RD and Vero cells (Supplementary Fig. S1c). Infected cells exhibited CPE characterized by cells rounding, and the presence of crescent-shaped nuclei with condensed chromatin due to CPE at 16 h post infection. The percentage of cells with crescent-shaped nuclei decreased after MOM treatment in a dose-dependent manner (Supplementary Fig. S1d,e). It was accompanied by diminished expression of EV71 proteins, including viral caspid (VP2), RNA dependent RNA polymerase (3D^p^°^l^), and protease (3CD^pr^°), in MOM-treated infected cells. Protein levels of 3D^p^°^l^ were reduced by 98% and 99%, respectively, in infected RD cells treated with 156 and 312 μg/ml of MOM (Fig. 1b). Expression levels of VP2 were lowered by more than 99% in infected cells treated with 156 and 312 μg/ml (Fig. 1b). The 50% inhibitory concentration (IC_50_) of MOM for inhibitory effect on EV71 in RD cells was 45.92 ± 1.05 µg/ml (Fig. 1c). These findings indicate that MOM possesses an anti-EV71 activity.

### Identification of antiviral constituents in MOM

**Figure 2:**
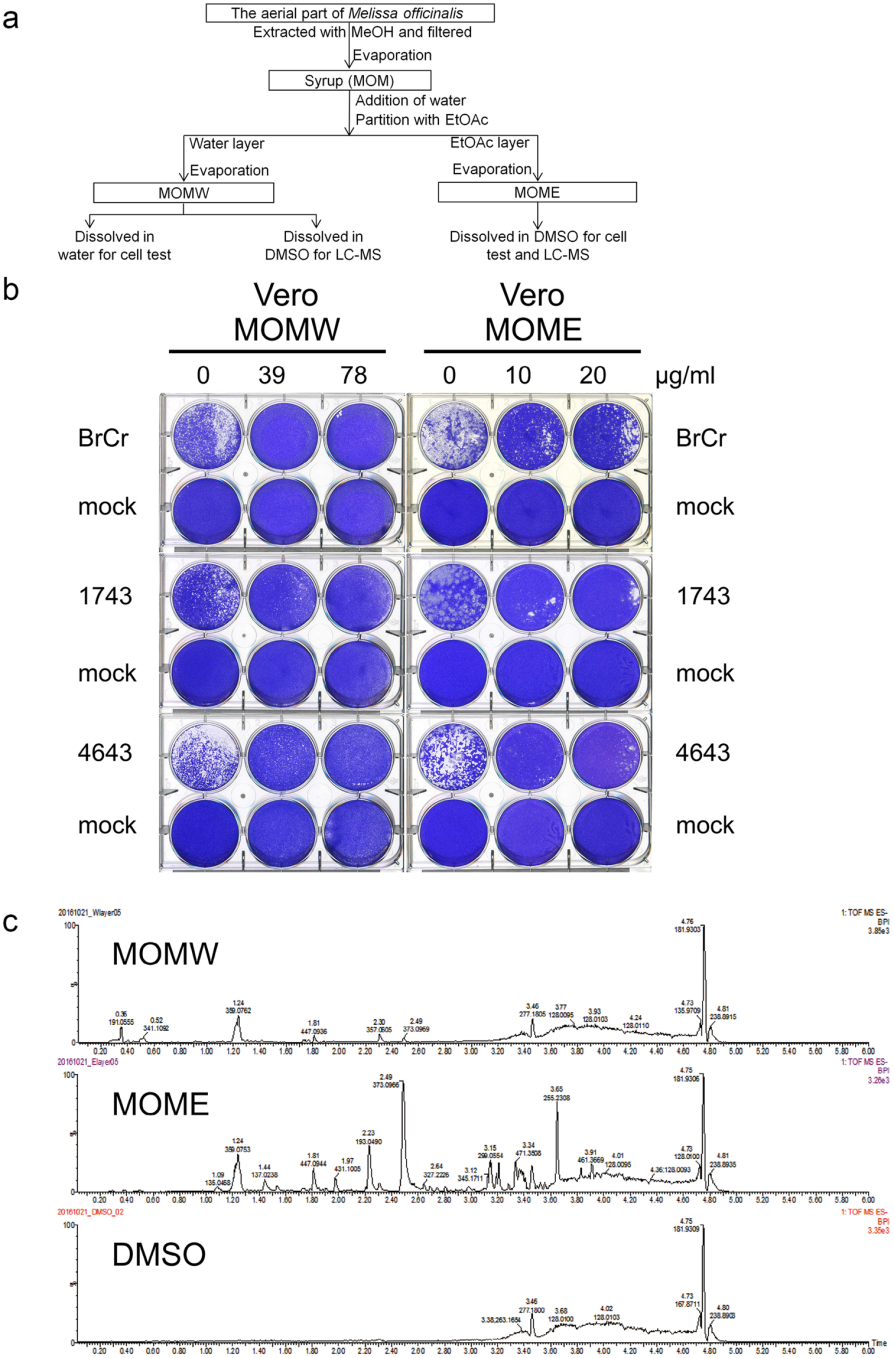
Identification of RA as an antiviral constituent of MOM. (**a**) The procedure for extraction and fractionation of MOM is shown in the flow chart. (**b**) Vero cells were mock- or infected with BrCr, 1743 and 4643 strains for 1 h, and were overlaid with 0.3% agarose in DMEM/2% FBS containing 0, 39, 78 μg/ml of MOMW or 0, 10, or 20 μg/ml of MOME. Vero cells were incubated for 5 days. Cells were fixed in 10% formalin and stained with 1% crystal violet solution. Representative plates are shown here. (**c**) MOMW and MOME, as well as the solvent DMSO were subject to UPLC-MS analysis. The base peak chromatogram is shown. The retention time and *m/z* for some of base peaks are shown. A representative experiment out of three is shown.

To identify the antiviral constituents in MOM, we diluted the dark green syrup of MOM in water and partitioned with equal volume of ethyl acetate (EtOAc) (Fig. 2a). The aqueous and organic fractions were evaporated, and the resulting fractions MOMW and MOME were obtained. These fractions were tested for their antiviral activity. Both MOMW and MOME fractions reduced plaque formation for all viral strains tested (Fig. 2b). At these concentrations, MOMW (CC_50_ = 153.7 ± 1.07 μg/ml, Supplementary Fig. S2a) and MOME (CC_50_ = 21.61 ± 1.08 μg/ml, Supplementary Fig. S2b) showed low cytotoxicity to Vero cells, as determined by standard neutral red assay. These findings suggest that these extracts have some biologically active components in common. The MOMW and MOME were dissolved in DMSO, and subject to liquid chromatography coupled with mass spectrometry (LC/MS) analysis. The base peak chromatogram is shown (Fig. 2c). The compound with mass-to-charge ratio (*m/z*) of 359.07 was most abundant in the analyte derived from aqueous fraction, and was present in similar quantity in the analyte derived from EtOAc fraction. The compound was further identified using LC/MS/MS (Supplementary Fig. S2c,e). The mass spectrum of this compound matched that of rosmarinic acid (RA) (Supplementary Fig. S2d,f). The compound was purified from MOMW using column chromatography (Supplementary Fig. S3a), and analyzed by nuclear magnetic resonance spectroscopy (NMR). The ^1^H-NMR spectrum of this compound (Supplementary Fig. S3b) was identical to that of RA (Supplementary Fig. S3c).

### RA inhibits EV71 replication

**Figure 3:**
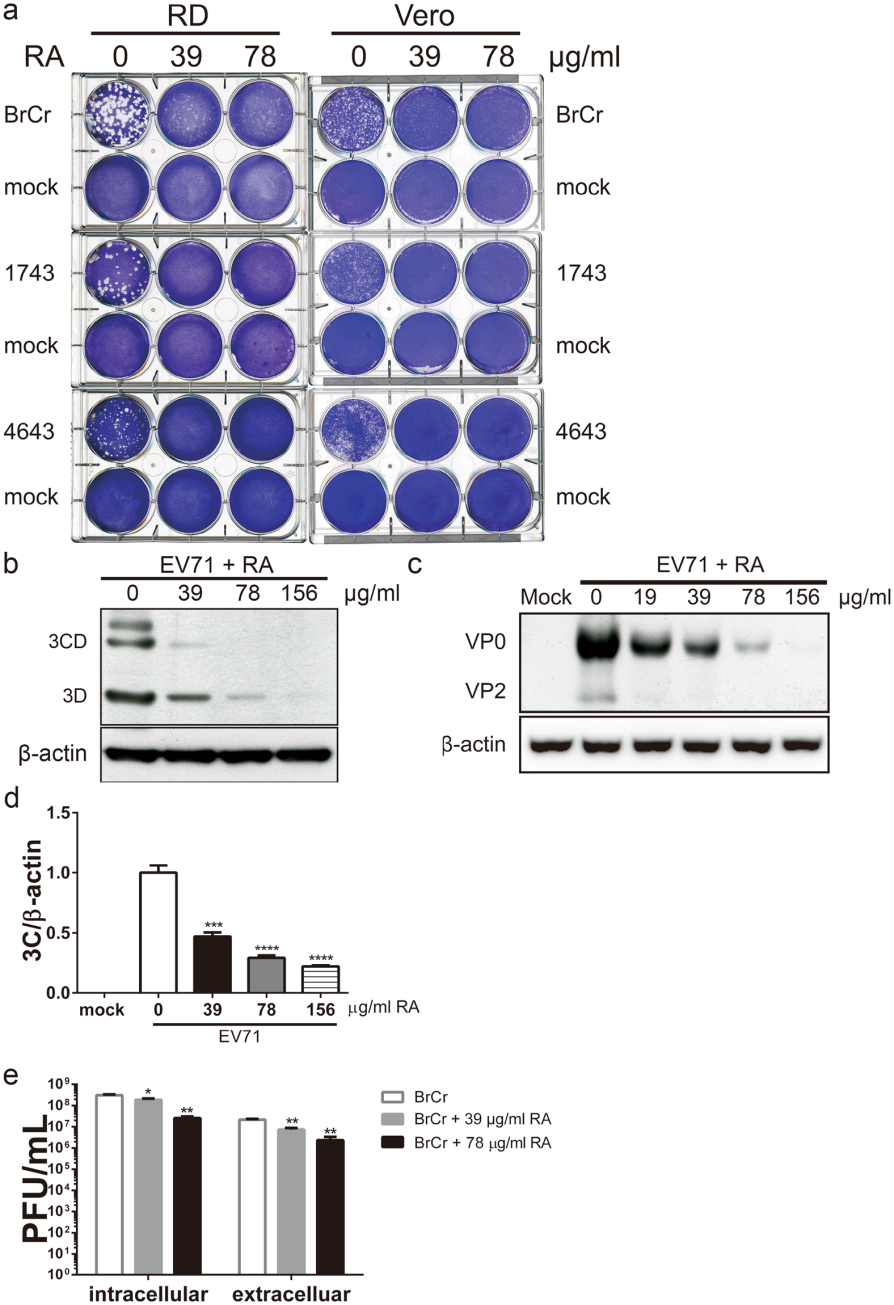
RA represses EV71 infection. (**a**) RD and Vero cells were mock- or infected with BrCr, 1743 and 4643 strains for 1 h, and were overlaid with 0.3% agarose in DMEM/2% FBS supplemented with 0, 39 or 78 μg/ml RA. The samples were processed as described in the legend of Fig. 1. Representative plates are shown here. (**b**) RD cells were infected EV71 at an m. o. i. of 0.05 in absence or presence of 39, 78 or 156 μg/ml RA for 16 h. Cellular protein was harvested, and was subject to western blotting with antibodies to 3D and β-actin. The cropped images of the blots are shown. The full-length blots are presented in Supplementary Fig. S9. A representative experiment out of three is shown. (**c**) RD cells were mock- or infected BrCr at an m. o. i. of 0.05 in the absence or presence of 19, 39, 78 or 156 μg/ml RA for 16 h. Cellular protein was harvested, and was subject to western blotting with antibodies to VP2 and β-actin. The cropped images of the blots are shown. The full-length blots are presented in Supplementary Fig. S10. A representative experiment out of three is shown. (**d**) RD cells were similarly infected as described in (**b**), and total RNA was isolated. The level of EV71 genomic copy was determined by quantitative reverse transcription PCR, and normalized to the level of β-actin. Data are expressed relative to that of untreated cells. The results are means ± SD of three separate experiments. ***P < 0.001, ****P < 0.0001 vs. infected cells without treatment. (**e**) RD cells were infected with BrCr at an m. o. i. of 5, and treated with 0, 39 or 78 μg/ml RA for 9 h. Extracellular and intracellular viral particles were harvested for plaque assay for titer determination. The results are means ± SD of three separate experiments. *P < 0.05, **P < 0.01, vs. infected cells without treatment.

Plaque formation was effectively inhibited by treatment with 39 or 78 μg/ml of RA for Vero and RD cells and for all viral strains tested (Fig. 3a). At these concentrations, RA was not cytotoxic to RD cells (CC_50_ = 209.8 ± 1.04 μg/ml, Supplementary Fig. S4a) and Vero cells (CC_50_ = 593.7 ± 1.08 μg/ml, Supplementary Fig. S4b). Consistent with this, expression of enteroviral protein 3D^p^°^l^ decreased by 78% and 91% in infected RD cells treated with 78 and 156 μg/ml RA (Fig. 3b). The levels of VP2 declined by 73% and 86%, respectively, in BrCr-infected cells upon treatment with 19 and 39 μg/ml RA (Fig. 3c and Supplementary Fig. S4c). The levels of VP0 were 42% and 63% lower in similarly treated BrCr-infected RD cells compared to cells infected alone. The discordant decrease in levels of these proteins implies that the processing of VP0 to VP2 may be affected. The ratio of the level of VP2 to that of VP0 decreases in MOM- or RA-treated cells in a dose dependent manner (Supplementary Fig. S4d) Additionally, RA inhibited synthesis of viral genomic synthesis in EV71-infected RD and Vero cells in a dose-dependent manner (Fig. 3d and Supplementary Fig. S4e). The level of EV71 RNA in infected RD cells decreased by 71% and 78%, respectively, upon treatment with 78 and 156 μg/ml RA (Fig. 3d). Similarly, its level in infected Vero cells declined by 79% after treatment with 312 μg/ml RA (Supplementary Fig. S4e). Decreases in viral RNA replication and protein synthesis is accompanied by reduction in progeny virus. Extracellular and intracellular viral particles decreased by 91% and 90% in infected RD cells upon treatment with 78 μg/ml RA (Fig. 3e). The IC_50_ of RA for inhibitory effect on EV71 in RD cells was 43.07 ± 1.05 μg/ml (Supplementary Fig. S4f). These findings validate the anti-EV71 activity of RA.

### RA inhibits EV71 infection at attachment and post-attachment stages

**Figure 4:**
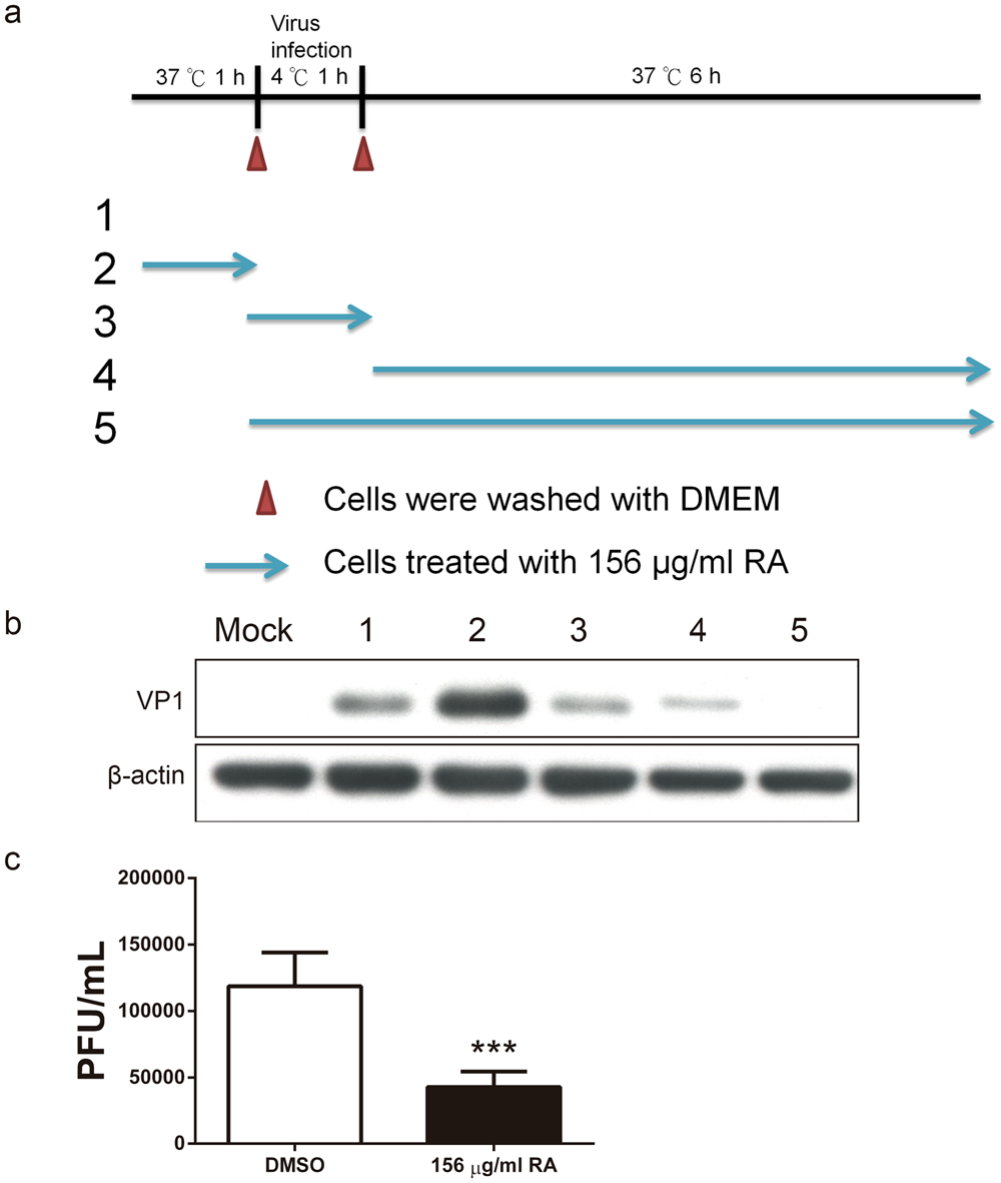
RA inhibits EV71 infection during viral adsorption and post-adsorption stages. (**a**) The time-of-addition experiment was performed to determine the phase of infection, at which RA exerts its antiviral activity. RD cells were mock-infected. RD cells were infected with BrCr at an m. o. i. of 20 at 4 °C for 1 h, washed free of unadsorbed virus, and cultured for 6 h prior to examination of VP1 expression (condition 1, positive control). Cells were pre-treated with 156 μg/ml RA, washed, incubated with virus, washed, and cultured for 6 h before analysis (condition 2). Cells were incubated with virus in the presence of 156 μg/ml RA at 4 °C for 1 h, washed, and cultured for 6 h at 37 °C before analysis (condition 3). Cells were incubated with virus at 4 °C for 1 h, washed, and cultured in the presence of 156 μg/ml RA at 37 °C for 6 h before analysis (condition 4). Cells were incubated with virus in the presence of 156 μg/ml RA at 4 °C for 1 h, washed, and treated with RA for 6 h at 37 °C before analysis (condition 5). (**b**) Expression of VP1 and β-actin in cells treated under aforementioned conditions was determined by western blotting. The cropped images of the blots are shown. The full-length blots are presented in Supplementary Fig. S11. A representative experiment out of three is shown. (**c**) Interaction between virions and RA reduces the viral infectivity. The viral particles were incubated with 156 μg/ml RA or 0.15% DMSO at 4 °C for 1 h. The reaction was filtered through filter, and the retentate was analyzed for viral titer. Results are means ± SD of three independent experiments. ****P < 0.001, vs. control treatment group.

As a first step in studying the antiviral mechanism of RA, we conducted a time-of-addition assay to identify the stages at which RA inhibits EV71 infection. RA was administered at different stages of BrCr infection (Fig. 4a), and expression level of viral protein VP1, indicative of viral replication, was studied. When cells were pre-treated with RA, RA failed to suppress EV71 infection, and slightly enhanced it (Fig. 4b, condition 2). Treatment of cells with RA during viral absorption (Fig. 4b, condition 3) and post-adsorption (Fig. 4b, condition 4) periods decreased viral replication moderately. Inclusion of RA during adsorption and post-absorption stages significantly reduced viral replication (Fig. 4b, condition 5). These findings indicate RA inhibits viral infection during viral attachment and after viral absorption. RA showed a synergic anti-EV71 effect when RA was present in both phases.

RA inhibits viral replication during viral attachment. It is possible that RA may interact with virions and prevent their interaction with cellular receptors. To test this hypothesis, viral particles were incubated with 156 μg/ml RA on ice for 1 h, and any excess of RA was removed using centrifugal filtration. The remaining viral titer was determined using plaque assay. Viral titer in RA-treated group was 4.28 ± 1.17 × 10^4^ PFU/ml, being lower than that of vehicle control group (1.18 ± 0.25 × 10^5^ PFU/ml) (Fig. 4c). Such finding suggests that RA may interact with virions, and interfere with their binding to cellular receptors.

### RA represses EV71-induced switch between cap-dependent and IRES-dependent translation

**Figure 5:**
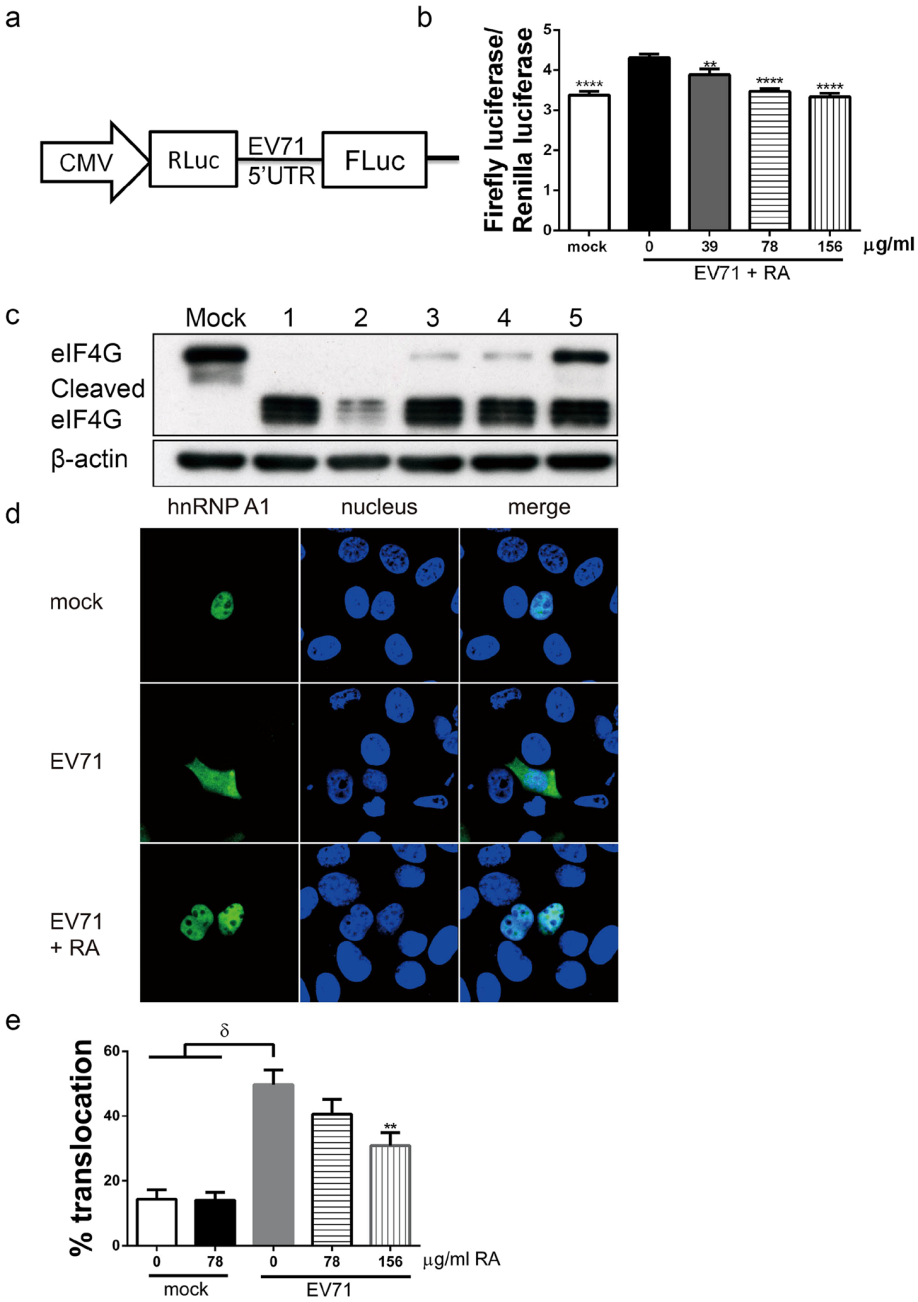
RA inhibits EV71-induced cessation of cap-dependent translation and initiation of IRES-dependent translation in host cells. (**a**) The bicistronic plasmid pRHF-EV71-5′UTR for analysis of cap-dependent and IRES-dependent translation is shown. CMV, cytomegalovirus promoter; Rluc, *Renilla* luciferase; Fluc, firefly luciferase. (**b**) RD cells were transfected with pRHF-EV71-5′UTR. Twelve hour later, the transfected cells were mock- or infected with BrCr at an m. o. i. of 20 for 1 h, and treated without or with 39, 78 or 156 μg/ml RA for 6 h. The activities of Fluc and Rluc were measured with Dual-Luciferase Reporter assay system. Results are means ± SD of three independent experiments. ****P < 0.001, **P < 0.01, vs. infected cells without treatment. (**c**) RD cells were infected with EV71 under conditions described in the legend of Fig. 4a. The condition of treatment is indicated by a number shown above the western blot image. Cells were harvested for western blotting with antibodies to eIF4G and β-actin. The positions of intact and cleaved eIF4G are indicated. The cropped images of the blots are shown. The full-length blots are presented in Supplementary Fig. S12. A representative experiment out of three is shown. (**d**) RD cells were transfected with expression vector encoding GFP-tagged hnRNP A1. After 48 hour later, the transfected cells were infected with EV71 at an m. o. i. of 20 for 1 h, and then treated without or with 156 μg/ml RA for 6 h. Infected cells were fixed, and stained with Hoechst 33342 and analyzed using confocal microscopy. A representative experiment out of three is shown. (**e**) Cells were transfected, infected, and treated with indicated concentrations of RA as described in (**d**). Cells were fixed, stained, and analyzed using IN Cell Analyzer 1000. The percentage of cells displaying cytoplasmic relocation of hnRNP A1 was quantified. Data are means ± SD of three independent experiments. δ, P < 0.0001, vs. mock-infected cells; **P < 0.01, vs. infected cells without treatment.

The ability of RA to inhibit viral replication during post-absorption phase prompts us to investigate the underlying antiviral mechanism. It is known that EV71 infection induces the switch between cap-dependent and IRES-dependent translation^20,23^. To study whether RA affects this molecular process, RD cells were transfected with a bicistronic plasmid pRHF-EV71-5′UTR (Fig. 5a), infected with BrCr for 1 h, and treated with RA. The ratio of firefly luciferase activity to *Renilla* luciferase activity (Fluc/Rluc) is indicative of the relative activities of IRES-dependent and cap-dependent translation. It was 27.66% higher in EV71-infected cells than in uninfected cells (p < 0.001) (Fig. 5b). RA mitigated such increase in Fluc/Rluc in a dose-dependent manner (Fig. 5b). It is possible that RA may disturb EV71-induced switch between cap-dependent and IRES-dependent translation. It is known that viral protease- 2A^pr^° hydrolyzes translation initiation factor eIF4G resulting in shutdown of cap-dependent translation^40^. To explore the possibility that RA treatment may inhibit EV71-induced eIF4G cleavage to block the shutdown of cap-dependent translation, we examined the expression level of eIF4G in EV71-infected cells. RD cells were infected with EV71 at a multiplicity of infection (m. o. i.) of 20, and treated with 156 μg/ml RA under conditions depicted in Fig. 4a. EV71 infection led to complete cleavage of eIF4G (Fig. 5c, condition 1). RA pre-treatment did not affect eIF4G cleavage (Fig. 5c, condition 2). When RA was added during either viral adsorption or post-adsorption phases, eIF4G cleavage was partially inhibited (Fig. 5c, condition 3 & 4). When RA was given during both viral adsorption and post-absorption phases, it acted synergistically to inhibit eIF4G cleavage (Fig. 5c, condition 5). These findings suggest that RA inhibits EV71-induced shutdown of cap-dependent translation through preservation of intact eIF4G.

We studied if RA inhibits IRES-dependent translation of enteroviral protein. Initiation of IRES-dependent translation is regulated by ITAFs, such as hnRNP A1. hnRNP A1 re-localizes to cytoplasm during infection, and interacts with IRES within EV71 5′UTR. To study the hypothesis that RA may interfere with this process, we transfected RD cells with an expression plasmid encoding a GFP-tagged hnRNP A1 (pGFP-hnRNP A1); infected the transfected cells with EV71; and analyzed the effect of RA on cytoplasmic translocation of GFP-tagged hnRNP. The GFP-tagged hnRNP A1 was localized to nuclei of mock-infected cells, and translocated from nuclei to cytoplasm in EV71-infected cells (Fig. 5d). Relocation of hnRNP A1 in infected cells was inhibited by treatment with 156 μg/ml RA (Fig. 5d). The percentage of cells showing cytoplasmic accumulation of GFP-tagged hnRNP A1 (i.e. cytoplasmic GFP-positive cells) was quantified using a high throughput imaging technique. The percentage of such cells was 14.39 ± 2.91% in mock-infected group, but it increased to 49.74 ± 4.52% in EV71-infected group (Fig. 5e). RA treatment decreased the percentage of cytoplasmic GFP-positive cells in a dose dependent manner. The percentage of infected cells showing hnRNP A1 relocation declined to 30.92 ± 3.97% upon treatment with 156 μg/ml RA (Fig. 5e). It is possible that RA may downregulate hnRNP A1 translocation and inhibit IRES-dependent translation.

### RA suppresses EV71-induced phosphorylation of p38 kinase

**Figure 6:**
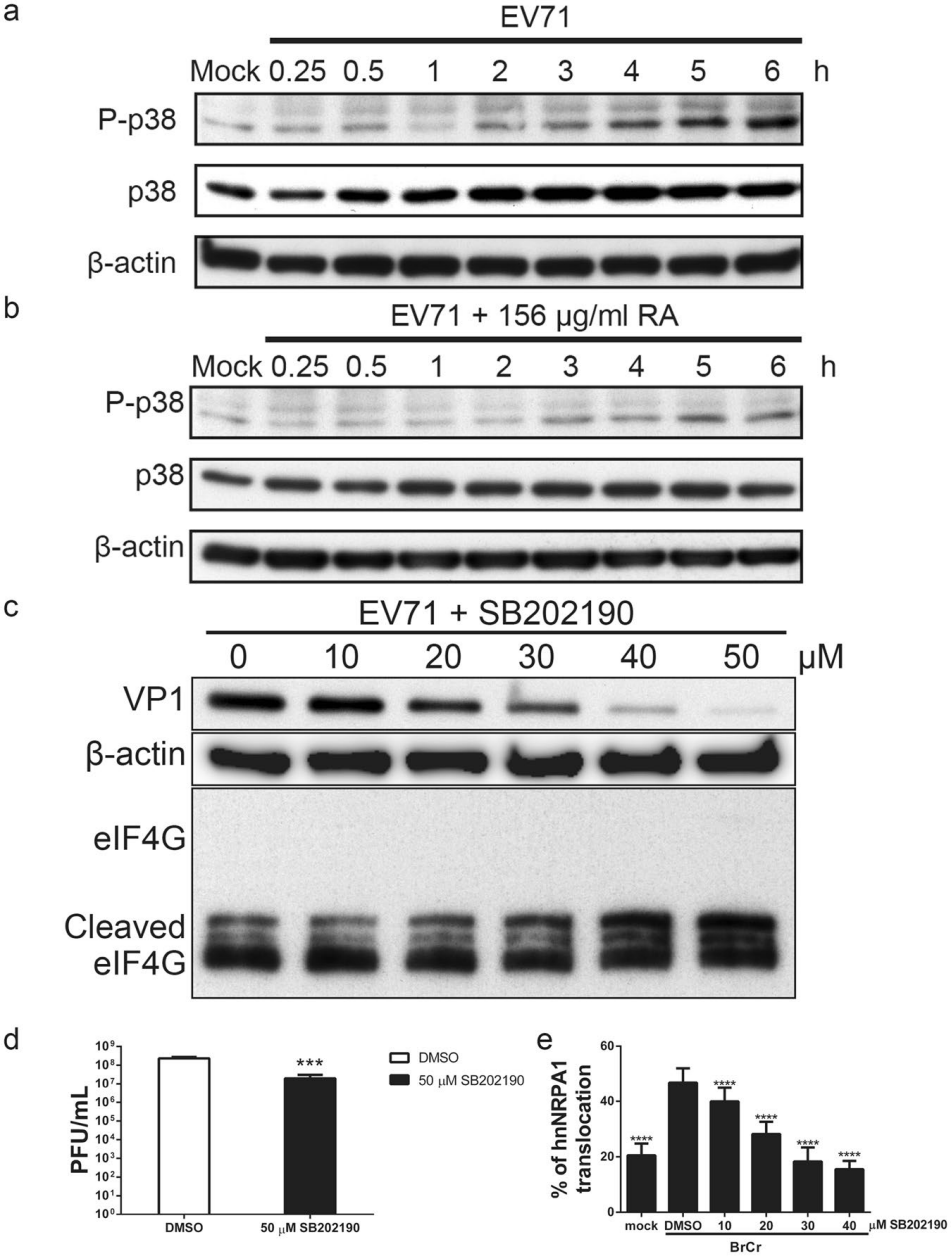
RA inhibits EV71-induced p38 kinase phosphorylation that is essential to EV71 infection process. (**a**,**b**) RD cells were mock- or infected with BrCr at an m. o. i. of 20 for 1 h, and subsequently treated without or with 156 μg/ml RA. The treated cells were harvested at 0.25, 0.5, 1, 2, 3, 4, 5 and 6 h. The phosphorylated p38 kinase (P-p38), and total cellular p38 (p38) was analyzed using western blotting. The blot was stripped, and incubated with antibody to β-actin. The cropped images of the blots are shown. The full-length blots are presented in Supplementary Figs S13 and S14. A representative experiment out of three is shown. (**c**) RD cells were pre-treated with 0.5% DMSO (vehicle control) or 10, 20, 30, 40 or 50 μM SB202190 for 1 h, and infected with BrCr at an m. o. i. of 5 for 1 h. The cells were harvested at 12 h p. i. for western blotting with antibodies to VP1, eIF4G and β-actin. The cropped images of the blots are shown. The full-length blots are presented in Supplementary Fig. S15. A representative experiment out of three is shown. (**d**) Cells were treated with vehicle or 50 μM SB202190 and infected as described in (**c**). The progeny virus was collected at 12 h p. i. for titer determination. Data are means ± SD of four separate experiments. ***P < 0.005, vs. vehicle control. (**e**) RD cells were transfected with hnRNP A1-GFP. Twenty-four hour later, the transfected cells were pre-treated with vehicle or 10, 20, 30 or 40 μM SB202190 for 1 h, and infected with BrCr at an m. o. i. of 20 for 1 h. Cells were fixed at 6 h p. i., stained with Hoechst 33342, and subject to image analysis using IN Cell Analyzer 1000. The percentage of cells showing cytoplasmic relocation of hnRNP A1 was quantified. Data are means ± SD of three separate experiments. ****P < 0.001, vs. infected cells treated with vehicle control.

Subcellular distribution of hnRNP A1 is regulated by p38 signaling^41^. The ability of RA to suppress EV71-induced hnRNP A1 redistribution raises the possibility that RA may regulate relocation of hnRNP A1 through its effect on p38 pathway. To test this hypothesis, we infected RD cells with BrCr at an m. o. i. of 20; treated the infected cells without or with 156 μg/ml RA; harvested their total protein for immunoblotting analysis of phosphorylated p38 kinase. EV71 infection induced biphasic phosphorylation of p38 kinase. Phosphorylation of p38 kinase slightly increased at around 15 to 30 min post infection. A stronger phosphorylation of p38 kinase in EV71-infected cells was observed during the period from 2 h to 6 h post infection (Fig. 6a). RA treatment repressed EV71-induced p38 phosphorylation (Fig. 6b). To demonstrate the role of p38 kinase in EV71 infection, RD cells were treated with p38 inhibitor SB202190, and infected with EV71. SB202190 inhibited expression of viral capsid protein VP1 in a dose-dependent manner (Fig. 6c). Production of progeny virus decreased by 92% in EV71-infected cells treated with 50 μM SB202190, as compared with that of vehicle control-treated cells (Fig. 6d). These results suggest that EV71-induced p38 phosphorylation can be suppressed by RA. Interestingly, the cleavage of eIF4G in EV71-infected cells was not altered upon SB202190 treatment (Fig. 6c), indicating that EV71-induced eIF4G cleavage is independent of p38 activation.

To study the causal relationship between p38 activity and hnRNP A1 relocation, we studied the effect of SB202190 on hnRNP A1 distribution. RD cells were transfected with pGFP-hnRNP A1, treated with SB202190, and infected with BrCr. SB202190 decreased EV71-induced relocation of hnRNP A1 in a dose-dependent manner (Fig. 6e). Such finding suggests that EV71 may induce cytoplasmic accumulation of hnRNP A1 via p38 signaling.

### RA suppresses p38 activation through an antioxidative mechanism

**Figure 7:**
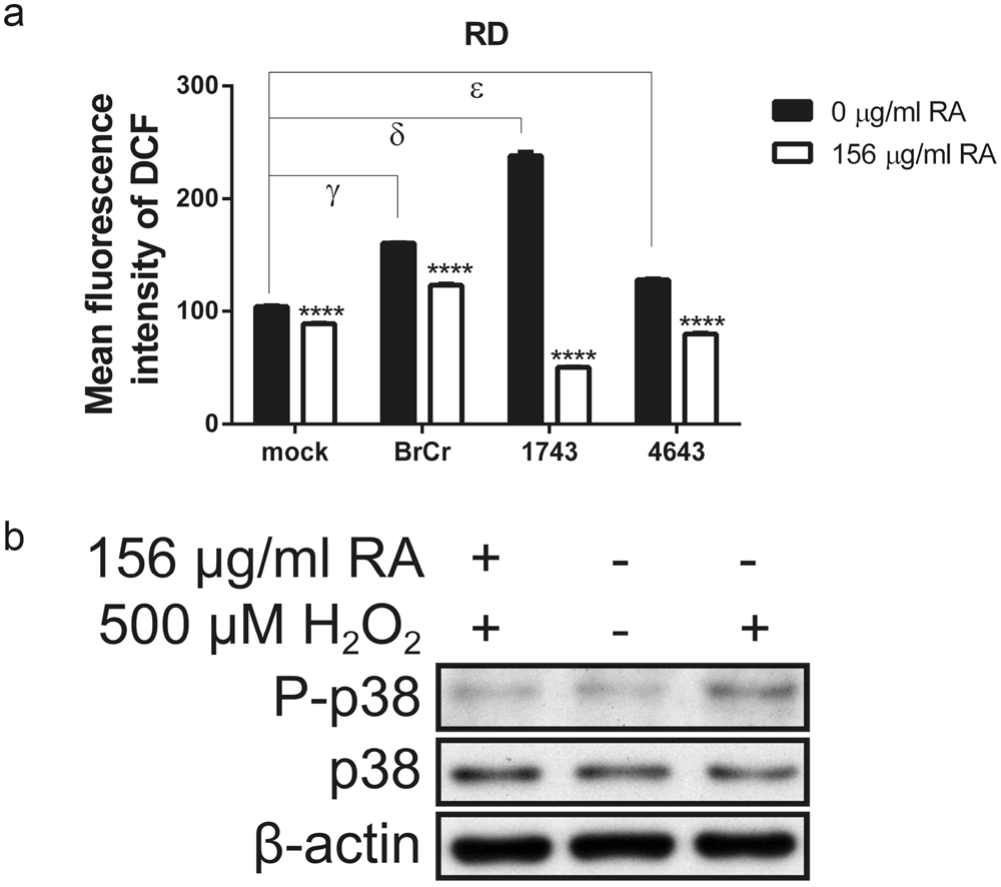
RA suppresses EV71-induced ROS generation and ROS-induced p38 kinase activation. (**a**) RD cells were mock- or infected with BrCr, 1743 or 4643 strains at an m. o. i. of 0.05 for 1 h, and subsequently treated with 156 μg/ml RA for 24 h. Cells were stained with H_2_DCFDA at 37 °C for 30 min, and analyzed using flow cytometry. Results are means ± SD of six separate experiments. ****P < 0.0001, vs. infected cells without treatment; γ, P < 0.0001, vs. BrCr-infected vs. mock-infected cells; δ, P < 0.0001, 1743-infected vs. mock-infected cells; ε, P < 0.0001, 4643-infected vs. mock-infected cells. (**b**) RD cells were treated without or with 500 μM H_2_O_2_ for 1 h in the absence or presence of 156 μg/ml RA. Levels of the phosphorylated and total form of p38 kinase and that of β-actin were examined by western blotting. The cropped images of the blots are shown. The full-length blots are presented in Supplementary Fig. S16. A representative experiment out of three is shown.

EV71 infection induces oxidative stress in host cells^28–30^, which activates phosphorylation of p38 kinase^42,43^. The antioxidant capacity of RA was determined using ferric reducing antioxidant power (FRAP) assay as 2.235 ± 0.035 g Trolox/g RA. It is hypothesized that RA scavenges EV71-induced ROS generation, and suppresses activation of p38. To study this hypothesis, we stained EV71-infected cells with H_2_DCFDA or CellROX Deep Red dye to determine the intracellular ROS generation. ROS levels increased in BrCr-, 1743-, and 4643-infected cells, as compared with that of mock-infected cells. RA treatment reduced ROS generation in EV71-infected cells (Fig. 7a and Supplementary Fig. S5a,b). To assess whether RA acts to block ROS-induced p38 activation, we treated RD cells with 500 μM H_2_O_2_, and examined the effect of RA on p38 phosphorylation. As expected, hydrogen peroxide induced p38 phosphorylation, but RA treatment mitigated p38 activation. Consistent with this, hydrogen peroxide induced cytoplasmic accumulation of hnRNP A1 in RD cells (Supplementary Fig. S5c). These results suggest that the ROS-induced p38 phosphorylation and hnRNP A1 translocation can be inhibited by RA.

### RA suppresses EV71-induced phosphorylation of epidermal growth factor receptor substrate 15 (EPS15)

**Figure 8:**
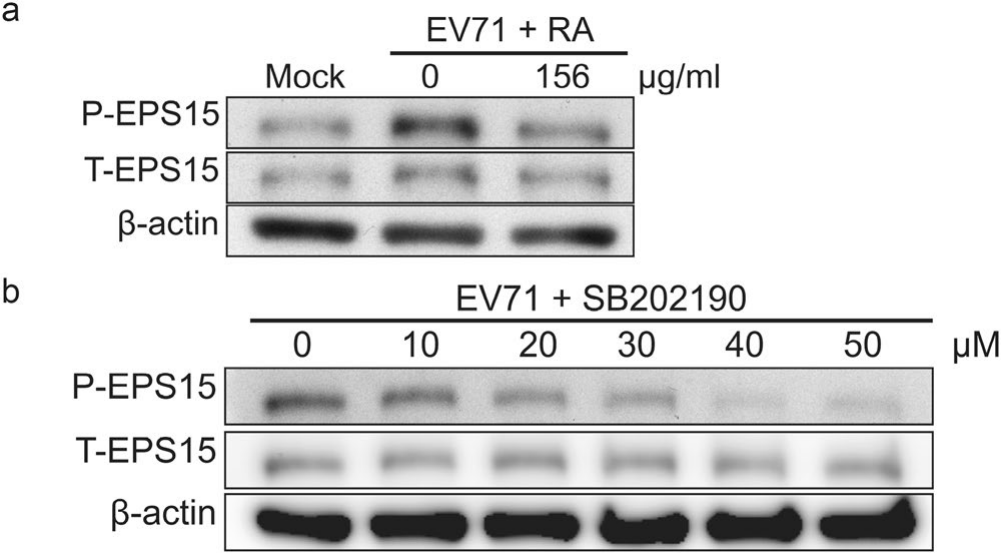
RA reduces EV71-induced phosphorylation of EPS15 at Ser796. (**a**) RD cells were mock- or infected with BrCr at an m. o. i. of 20 for 1 h, and treated without or with 156 μg/ml RA for 7 h. Cells were harvested and subject to western blotting with antibodies to β-actin, EPS15 and EPS15 phosphorylated at Ser796. The cropped images of the blots are shown. The full-length blots are presented in Supplementary Fig. S17. A representative experiment out of three is shown. (**b**) RD cells were pre-treated with 0.5% DMSO (vehicle control) or 10, 20, 30, 40, or 50 μM SB202190 for 1 h, and infected with BrCr at an m. o. i. of 5 for 1 h. Cells were harvested at 12 h p. i. for western blotting as described in (**a**). The cropped images of the blots are shown. The full-length blots are presented in Supplementary Fig. S18. A representative experiment out of three is shown.

EPS15, a target protein of p38^44^, regulates intracellular trafficking^45^, which is essential to viral replication^46^. EV71 infection led to increase in phosphorylation of EPS15 at Ser796. RA treatment significantly reduced such increase in infected RD cells (Fig. 8a, lane 2 & 3). To address whether p38 induces EPS15 phosphorylation, RD cells were treated with SB202190, infected with EV71, and EPS15 phosphorylation was detected using immunoblotting. SB202190 significantly reduced EV71-induced phosphorylation of EPS15 in a dose-dependent manner (Fig. 8b). These results suggest that EV71 induces EPS15 phosphorylation through p38 kinase pathway. It is possible that RA can inhibit EPS15 phosphorylation by suppressing p38 signaling.

### RA protects mice from EV71 infection

To study whether RA protects mice from EV71 infection, seven-day-old specific pathogen-free ICR mice were infected with mouse-adapted virus intraperitoneally, and were subsequently treated with RA (50 mg/kg) or PBS at daily intervals for 14 day. The survival rate of RA-treated mice (66.67%, n = 6) were higher than that of PBS-treated mice (33.33%, n = 6) (Supplementary Fig. S6a). Moreover, a higher percentage of RA-treated mice were rehabilitated from illness than PBS-treated mice (Supplementary Fig. S6b,c). These results suggest RA may offer protection against EV71 infection *in vivo*.

## Discussion

The present study has shown that the MOM exhibits anti-EV71 activity. RA, identified as an antiviral constituent of MOM, inhibits viral replication, and offers protective effect against EV71 infection *in vivo*. Mechanistically, RA suppresses attachment of virion to host cells, eIF4G cleavage, and cytoplasmic relocation of hnRNP A1. RA acts as ROS scavenger to block ROS signaling and p38 kinase activation, resulting in diminished cytoplasmic hnRNP A1 relocation and EPS15 phosphorylation. Our findings suggest that RA exerts its antiviral effect via multiple mechanisms.

MO originates from Southern Europe, Mediterranean region, Middle East countries and North Africa and is cultivated worldwide now^32^. MO is commonly consumed in food and beneficial drinks. Choi *et al*. have shown that the MO extract prepared by extraction in water at 40 °C possesses antiviral activities^39^. MO extract prepared by methanolic extraction at 60 °C has shown an anti-EV71 activity in our study. However, methanolic extraction at room temperature yielded an extract with no anti-EV71 activity in Choi’s study^47^. A major difference may lie in the temperature at which the extraction was performed. The higher extraction temperature employed in our study may be beneficial to extraction of biologically active antiviral constituents from MO. The study of the antiviral mechanism of MOM necessitates identification of biologically active constituents. The MOM diluted in water was further partitioned with an equal volume of EtOAC. Both layers were further processed, and the resulting fractions retained anti-EV71 activity (Fig. 2). Using ultra performance liquid chromatography coupled with mass spectrometry (UPLC-MS) as well as nuclear magnetic resonance (NMR) spectroscopy, we found that RA was present in both aqueous and organic fractions. Chemically speaking, RA is an ester of caffeic acid and 3,4-dihydroxyphenylacetic acid^48^. It is found in many plants within Labiatae family^49^. Several plants of this family, including *Salvia miltiorrhiza* (danshen)^50^ and *Ocimum basilicum* ^51^, show anti-EV71 activities. Rosmarinic acid has also been identified as an antiviral constituent of *Salvia miltiorrhiza* ^52^. Our finding that RA had antiviral activity against a number of clinical relevant EV71 strains, namely 1743 and 4643 strains, is interesting (Fig. 3a). This raises the hope of developing a therapeutic against EV71, and possibly other neurotropic viruses.

A time-of-addition assay has been used to examine the mechanistic stages at which RA acts to inhibit EV71 infection process. RA acts during viral adsorption and post-adsorption phases (Fig. 4b). Incubation of virus with RA reduced its infectivity (Fig. 4c), implying that RA may bind directly to enteroviral particles and interfere with their attachment to cellular receptor. It is known that MO extract and RA can directly interact with enveloped viral particles. The density of HIV-1 virion increases after treatment with an aqueous MO extract^36^, possibly as a consequence of chemical modification of viral particle by and binding of extract constituents. Additionally, MO extract and RA inhibit attachment of herpes simplex virus type I^35^. It remains elusive how MO constituents and RA act to interfere with viral attachment, and whether a similar mechanism is involved for enveloped viruses and non-enveloped enterovirus.

Several mechanisms may be held accountable for the anti-EV71 activities of RA at post-adsorption phase. RA suppresses the EV71-induced subversion of host cell translation and initiation of viral translation. Cleavage of eIF4G and PABP by 2A^pr^° and that of PABP by 3C^pr^° are implicated in this process^24^. It is probable that RA inhibits the viral proteases. For instance, 2A^pr^° is a cysteine protease homologous to the trypsin-like family of serine protease^53^. RA can react with the active site of another cysteine protease caspase 3^54^. It is possible that RA may inhibit viral proteases in a similar way. Additionally, RA may suppress viral translation and reduce the expression of viral proteases. Initiation of viral translation involves association of IRES-specific trans-acting factors (ITAFs) and ribosomal subunits with type I IRES^19,20^. An ITAF, hnRNP A1, relocates from nucleus to cytoplasm during infection, and binds to stem loops II and VI of EV71 IRES to enhance translational initiation^21,22^. The inhibitory effect of RA on hnRNP A1 translocation may lead to reduction in viral translation and replication. Interestingly, the discordance in the expression levels of VP0 and VP2 implies that the processing of VP0 is adversely affected by MOM and RA. It is probable that RA and biologically active constituents of MOM may inhibit viral protease and VP0 cleavage.

Subcellular distribution of hnRNP A1 can be regulated by ROS. Arsenite that induces oxidative stress causes hnRNP A1 relocation to cytoplasm^55–57^. We have also found that treatment of RD cells with 500 μM hydrogen peroxide induces cytoplasmic relocation of hnRNP A1 (Supplementary Fig. S5c). EV71 infection is known to instigate mitochondrial ROS generation and/or NADPH oxidase activation^28,29^. The increase in ROS may activate downstream signaling and hnRNP A1 translocation. Given the potent antioxidative capacity of RA (Fig. 7a), it may scavenge ROS to block such molecular events.

Activation of MAPK-related pathways has been implicated in pathogenesis of EV71^58,59^. Increased phosphorylation of p38 kinase was observed during the early (15 min to 30 min) and late (2 to 6 h p. i.) stages of EV71 life cycle in RD cells (Fig. 6). It is likely that p38 kinase activation is essential to EV71 replication. Pharmacological inhibition of p38 kinase significantly reduced viral translation (Fig. 6e). Consistent with this, p38 kinase signaling cascade plays a regulatory role in subcellular distribution of hnRNP A1^41,58^. Activation of this pathway enhances hnRNP A1 relocation, while its inhibition thwarts the process^41^. In agreement with this, we have shown that treatment with p38 kinase inhibitor blocked EV71-induced cytoplasmic relocation of hnRNP A1 (Fig. 6e). Our finding that RA treatment suppressed EV71-induced activation of p38 kinase is intriguing (Fig. 6). It implies that RA acts on p38 kinase or upstream of it. It has been shown that ROS can activate p38 kinase cascade^60,61^. Hydrogen peroxide treatment induced phosphorylation of p38 kinase, which was inhibited in the presence of RA (Fig. 7b). As aforementioned, RA acts as ROS scavenger, and in this manner, may prevent p38 kinase activation. It is not unprecedented. It has been shown that antioxidant treatment suppresses ROS-induced p38 kinase activation^61^. Additionally, RA is known to regulate the expression of antioxidative enzymes. For example, administration of RA to aging mice induces expression of superoxide dismutase, catalase and glutathione peroxidase in their liver and kidney^62^. These findings suggest that RA may repress activation of p38 kinase and translocation of hnRNP A1 through an antioxidative mechanism.

EPS15, another target of p38 kinase, plays an important role in EV71 infection^63^. EPS15 co-localizes with α-adaptin and clathrin^64^, and is believed to modulate clathrin-mediated endocytosis (CME) and membrane trafficking^45^. Recent findings have suggested that phosphorylation regulates the biochemical activity of EPS15^45^. EPS15 can be phosphorylated by p38 at Ser796^44^, and this phosphorylation site is implicated in regulation of endocytic process. Silencing of EPS15 has a suppressive effect on enteroviral infection^63^. EPS15 may be involved in CME of virus, and in membrane trafficking essential to EV71 replication. It has been recently shown that enterovirus utilizes the endocytic machinery to distribute cholesterol to replication organelles, where cholesterol facilitates the replication and 3CD^pr^° processing^46^. The antiviral activity of RA may be in part accounted for by its ability to inhibit p38 kinase and EPS15 phosphorylation.

Based on our findings, we propose a model for antiviral activity of RA (Supplementary Fig. S7). RA acts in multiple ways to inhibit viral replication. RA binds to viral particles to interfere with their attachment to receptors and internalization; suppresses eIF4G cleavage; removes ROS and inhibits activation of p38 kinase, blocking hnRNPA1 translocation and EPS15 phosphorylation.

## Methods

### Plant material

Dried aerial parts of *Melissa officinalis* were authenticated and supplied by Lian-Tung Trading Co. (Tihua St, Taipei, Taiwan). A voucher specimen (CGU-MO-1) was deposited in the herbarium of Chang Gung University, Taoyuan, Taiwan.

### Chemicals and reagents

Unless otherwise stated, all chemicals were purchased from Sigma-Aldrich (St. Louis, MO, USA). Acetonitrile (ACN), methanol (MeOH, UPLC grade), water (UPLC grade), ethyl acetate (EtOAc, LC-MS CHROMA SLOV grade), dimethyl sulfoxide (DMSO, Cell culture grade), Sephadex LH-20 (particle size 25–100 μm), Diaion HP-20 (particle size 250–850 μm), SB202190, crystal violet and RA were purchased from Sigma-Aldrich (St. Louis, MO, USA). Dulbecco’s modified Eagle’s medium (DMEM), Modified Eagle’s medium (MEM), OPTI-MEM, penicillin (10000 U/ml) and streptomycin (10000 μg/ml) (P/S), L-glutamine (200 mM), MEM non-essential amino acids solution (NEAA), MEGAscript T7 Transcription Kit, ReverseAid^TM^ First cDNA Synthesis Kit, MluI, Lipofectamine 2000, CellROX Deep Red reagents, 2′, 7′-dichlorodihydrofluorescein diacetate (H_2_DCFDA) and Hoechst 33342 were available from Thermo Fisher Scientific Inc. (Waltham, MA, USA). Fetal bovine serum (FBS) was obtained from Caisson Laboratories, Inc. (East Smithfield, UT, USA). The SsoFast EvaGreen supermix was purchased from Bio-Rad Laboratories, Inc (Hercules, CA, USA). The agarose was obtained from Amresco Inc. (Solon, OH, USA).

### Extraction, purification, and analytical procedures of MO

Dried leaves and stems of MO (500 g) were extracted with MeOH (5 L) at 60 °C for 4 hours twice. Crude extract of MO was filtered through Waterman No. 1 filter paper (GE Healthcare Life Sciences; Chicago, IL, USA). The liquid fraction was concentrated with a rotary evaporator to give dark green syrup named MOM (with a weight of 94.33 g). MOM was diluted in water and further partitioned with equal volume of EtOAc. The water and EtOAc layers were separated, and concentrated with a rotary evaporator to give dark brown concentrate (MOMW) and dark green powder (MOME), respectively. MOMW was diluted in water for cell studies and in DMSO for chemical analysis. MOME was dissolved in DMSO. The MOMW, MOME and solvent DMSO were chromatographed on an ACQUITY UPLC BEH C18 column (1.7 μm, 2.1 × 100 mm, Waters, USA) using a gradient of 5% ACN to 50% ACN. Each fraction was subject to electrospray ionization tandem mass spectrometry (ESI-MS/MS; HDMS-G1, Waters, USA) in negative ion mode. For purification of biologically active compounds of MOMW, the syrup was diluted with water. It was subject to chromatography on Diaion HP-20 column, followed by chromatography on Sephadex LH-20 column. The eluents were water, water/methanol (1:1, V/V) and methanol. The eluate MOMW-1-1 was further evaporated to obtain a light-brown syrup. The sample was analyzed by proton nuclear magnetic resonance spectroscopy (Bruker Avance III-400 NMR spectrometer; Bruker Daltonik GmbH, Bremen, Germany).

### Cells and viruses

Human rhabdomyosarcoma cells (RD; ATCC CCL-136) were maintained in DMEM supplemented with 10% FBS, 100 units/ml penicillin, 0.1 mg/ml streptomycin, 2 mM L-glutamine, and 0.1 mM NEAA. Cells were cultured at 37 °C in a humidified environment of 5% CO_2_. African green monkey kidney epithelial cells (Vero; ATCC CCL-81) were cultured in MEM with 10% FBS, 100 U/ml penicillin, and 0.1 mg/ml streptomycin. Three clinical isolated EV71 strains were used. The prototype strain of EV71, CA-BrCr-70 (BrCr), belongs to genogroup A. Clinical isolate TW-1743-98 (1743) belongs to genogroup B4^65^, while Tainan/4643/TW (4643) is classified in genogroup C2^66^. Virus stocks were propagated in Vero or RD cells as previously described^30^. Mouse adapted EV71 strain, MP4, was prepared from an infectious clone, MP4/y5^67^. Infectious clone was digested by MluI, and viral RNA was prepared using the MEGAscript T7 Transcription Kit (Thermo Fisher Scientific, Waltham, MA, USA). RD cells were set in 6-well plates at 4 × 10^5^ per well and incubated overnight. Three microgram of viral RNA was transfected into RD cell using lipofectamine 2000 (Thermo Fisher Scientific, Waltham, MA, USA) according to the manufacturer’s instructions. Virus particles were harvested at 24 h p. i. in three freeze-thaw cycles. The MP4 virus was further propagated in RD cells once before animal study. The titer of resulting viral stock was determined by plaque assay.

### Viral plaque assay and modified plaque assay screening for antiviral activity

The titer of virus was determined by plaque assays with Vero or RD cells. The viral supernatant was serially diluted with serum free medium in a ten-fold manner. The monolayer cells at a confluence of 80% were infected with diluted viral supernatants, and the viral titer was quantified in the form of plaque forming unit (PFU) per ml as previously described^30^.

For plaque reduction assay, RD and Vero cells in six-well plates were respectively infected with 100 and 200 PFU of virus for 1 h at 37 °C. After removal of unabsorbed virus, cells were overlaid with 0.3% agarose in medium supplemented with 2% serum and an indicated concentration of extract or RA. In the control group, mock infected cells were treated with the same concentrations of test samples. The plaque size and number were determined for assessment of the antiviral activity of extract or RA.

### Determination of the genomic copy number of EV71

For quantification of intracellular viral RNA, total cellular RNA was extracted from EV71-infected cells using TRIzol reagent (Thermo Fisher Scientific, USA) according to the manufacturer’s instructions. Concentration of total cellular RNA was determined by spectrophotometer (Nanophotometer, Implen, Germany). The relative copy number of EV71 was quantified by quantitative reverse transcription polymerase chain reaction (qRT-PCR) as previously described^30^.

### Infectivity inhibition assay

EV71 (BrCr) was diluted to 1 × 10^5^ PFU/ml in 10 ml, and incubated with or without 156.25 μg/ml RA for 1 h on ice. The medium containing unadsorbed virus was transferred to Amicon Ultra centrifugal filter unit with Ultracel-100 membrane (#UFC910024, Merck Millipore Darmstadt, Germany), and RA was removed by centrifugation at 5000 × g at 4 °C for 30 min. Viral particles in the retentate were resuspended in 1 ml serum-free DMEM. The titer of virus was quantified by plaque assay.

### Bicistronic reporter assays for detection of IRES activity

The biscistronic construct pRHF-EV71-5′UTR contains 5′UTR region of EV71 between *Renilla* luciferase (Rluc) and firefly luciferase (Fluc) genes, and has been used for assay of IRES activity^68^. The plasmid DNA (0.5 µg) was transfected into RD cells with Lipofectamine 2000 according to manufacturer’s protocols. Twelve hours after transfection, cells were un- or infected with 6 × 10^6^ PFU of virus for an hour. One hour later, DMEM/2% FBS supplemented with indicated final concentrations of RA was added to each well. After 6 h, cell lysates were harvested and activities of Rluc and Fluc were measured with dual-luciferase reporter assay system (Promega, Madison, USA) and GloMax 20/20 single tube luminometer (Promega, Madison, USA) according to the manufacturer’s instructions.

### hnRNP A1 translocation assay

To assess translocation of nuclear protein hnRNP A1, we employed the construct pGFP-hnRNP A1, which encodes a green fluorescent protein (GFP) tagged hnRNP A1 protein. RD cells were transfected with 0.5 µg of pGFP-hnRNP A1 plasmid using Lipofectamine 2000. Two days later, the cells were infected with or without 6 × 10^6^ PFU BrCr in serum-free medium at 37 °C for 1 h. After virus adsorption, DMEM containing 2% FBS and 78 or 156 μg/ml of RA was added, and the infected cells were incubated for another 6 h at 37 °C. Infected and mock treated cells were fixed with 10% formalin for 30 min, and the cell nuclei were stained with Hoechst 33342 (Thermo Fisher Scientific Inc., Waltham, MA, USA) in PBS at room temperature. The fluorescent images were obtained using IN Cell Analyzer 1000 (GE Healthcare Life Sciences, USA). The region of nucleus was defined as a blue fluorescent region. The translocation of hnRNP A1 from nucleus to cytoplasm is visualized as an increase in the intensity of green fluorescence in cytoplasm over that in nucleus. The percentage of cells showing hnRNP A1 relocation was quantified from 30 random fields per well.

For confocal microscopic study, RD cells were cultured in poly-D-lysine-coated 35 mm glass bottom culture dish (MatTek Corporation, MA, USA), and treated as described above. The cells were analyzed with Zeiss LSM 780 system (Carl Zeiss Microimaging GmbH, Heidelberg, Germany) as previously described^30^.

### Western blotting

Cellular lysate was separated by SDS-PAGE and analyzed by western blot as previously described^28^. Antibodies used in this study were listed in Supplementary Table S1.

### Ferric reducing/antioxidant power (FRAP) assay

The antioxidant power of RA was measured as previously described^69^. This method is based on the ability of antioxidant to reduce ferric-tripyridyltriazine complex to the blue-colored ferrous form, which has an absorption maximum at 593 nm. The absorbance was analyzed by ELISA reader (VersaMax, Molecular Devices LLC, CA, USA).

### Detection of Cellular Reactive Oxygen Species (ROS)

For determination of the intracellular ROS formation, cells were stained with cell-permeable fluorogenic dyes, CellROX Deep Red reagent or H_2_DCFDA as previously described^28,30^.

### Ethic statement

All animal methods and care described in the present study were carried out in accordance with national guide. They were approved by the Institutional Animal Care and Use Committee of Chang Gung Memorial Hospital at Linkou (IACUC No. 2012101801).

### Statistical analyses

Results are presented as mean ± SD. Statistical calculations were performed with Graphpad Prism 5 software (GraphPad Software Inc., San Diego, California, USA). Data were analyzed by Student’s *t*-test. A *p* value of less than 0.05 is considered significant.

## Electronic supplementary material

Supplementary information
