# Supplementary material for: Anti-enterovirus 71 activities of Melissa officinalis extract and its biologically active constituent rosmarinic acid
Source: Sci Rep. 2017 Sep 25;7:12264. doi: 10.1038/s41598-017-12388-2 (PMC5613005; doi:10.1038/s41598-017-12388-2)
Supplement: Supplementary file 1 — Supplementary information [file 41598_2017_12388_MOESM1_ESM.pdf]

Anti-enterovirus 71 activities of *Melissa officinalis* extract and its biologically active constituent rosmarinic acid

Authorship

Sin-Guang Chen[1], Yann-Lii Leu[2,3,4], Mei-Ling Cheng[5,6,7,8], Siew Chin Ting[9], Ching-Chuan Liu[10,11], Shulhn-Der Wang[12], Cheng-Hung Yang[1], Cheng-Yu Hung[6,7], Hiroaki Sakurai[13], Kuan-Hsing Chen[14], Hung-Yao Ho[6,8,9,*]

[1]Graduate Institute of Biomedical Science, Chang Gung University, Guishan, Taoyuan, Taiwan

[2]Graduate Institute of Natural Products, College of Medicine, Chang Gung University, Taoyuan, Taiwan

[3]Center for Traditional Chinese Medicine, Chang Gung Memorial Hospital at Linkou, Guishan, Taoyuan, Taiwan

[4]Chinese Herbal Medicine Research Team, Healthy Aging Research Center, Chang Gung University, Taoyuan, Taiwan

[5]Department of Biomedical Sciences, College of Medicine, Chang Gung University, Guishan, Taoyuan, Taiwan

[6]Healthy Aging Research Center, Chang Gung University, Guishan, Taoyuan, Taiwan

[7]Metabolomics Core Laboratory, Chang Gung University, Guishan, Taoyuan, Taiwan

[8]Clinical Phenome Center, Chang Gung Memorial Hospital at Linkou, Guishan, Taoyuan, Taiwan

[9]Department of Medical Biotechnology and Laboratory Science, College of Medicine, Chang Gung University, Taoyuan, Taiwan

[10]Department of Pediatrics, National Cheng Kung University Hospital, College of Medicine, National Cheng Kung University, Tainan, Taiwan

[11]Center of Infectious Disease and Signaling Research, National Cheng Kung University, Tainan, Taiwan

[12]School of Post-Baccalaureate Chinese Medicine, College of Chinese Medicine, China Medical University, Taichung, Taiwan

[13]Department of Cancer Cell Biology, Graduate School of Medicine and Pharmaceutical Sciences, University of Toyama, Toyama, Japan

[14]Kidney Research Center, Chang Gung Memorial Hospital, Chang Gung University, School of Medicine, Taoyuan, Taiwan

*Correspondence and requests for materials should be addressed at H.-Y. H.

Supplementary Table S1. Antibodies used in this study

Product name	Cat. No.	Provider or manufacturer
Mouse monoclonal anti-enterovirus 71 antibody	MAB979	Merck Millipore (Darmstadt, Germany)
Mouse monoclonal anti-β-actin antibody	A5441	Merck Millipore (Darmstadt, Germany)
Rabbit polyclonal anti-β-actin antibody	A2103	Merck Millipore (Darmstadt, Germany)
Rabbit polyclonal anti-p38 MAPK antibody	9212	Cell Signaling Technology (Danvers, MA, USA)
Rabbit polyclonal anti-phospho-p38 MAPK antibody	9211	Cell Signaling Technology (Danvers, MA, USA)
Rabbit polyclonal anti-EV71-VP1 antibody	PAB7631-D01P	Abnova (Neihu District, Taipei City, Taiwan)
Rabbit polyclonal anti-eIF4G3 antibody	GTX118109	GeneTex Inc. (Irvine, CA, USA)
Rabbit polyclonal anti-phospho-Ser-796-Eps15 antibody		Prof. Hiroaki Sakurai
Mouse monoclonal anti-EV71-3D antibody		Prof. Shin-Ru Shih
Goat anti-rabbit IgG-HRP antibody	sc-2004	Santa Cruz Biotechnology (Dallas, TX, USA)
Goat anti-mouse IgG-HRP antibody	sc-2005	Santa Cruz Biotechnology (Dallas, TX, USA)
Donkey anti-goat IgG HRP antibody	sc-2056	Santa Cruz Biotechnology (Dallas, TX, USA)

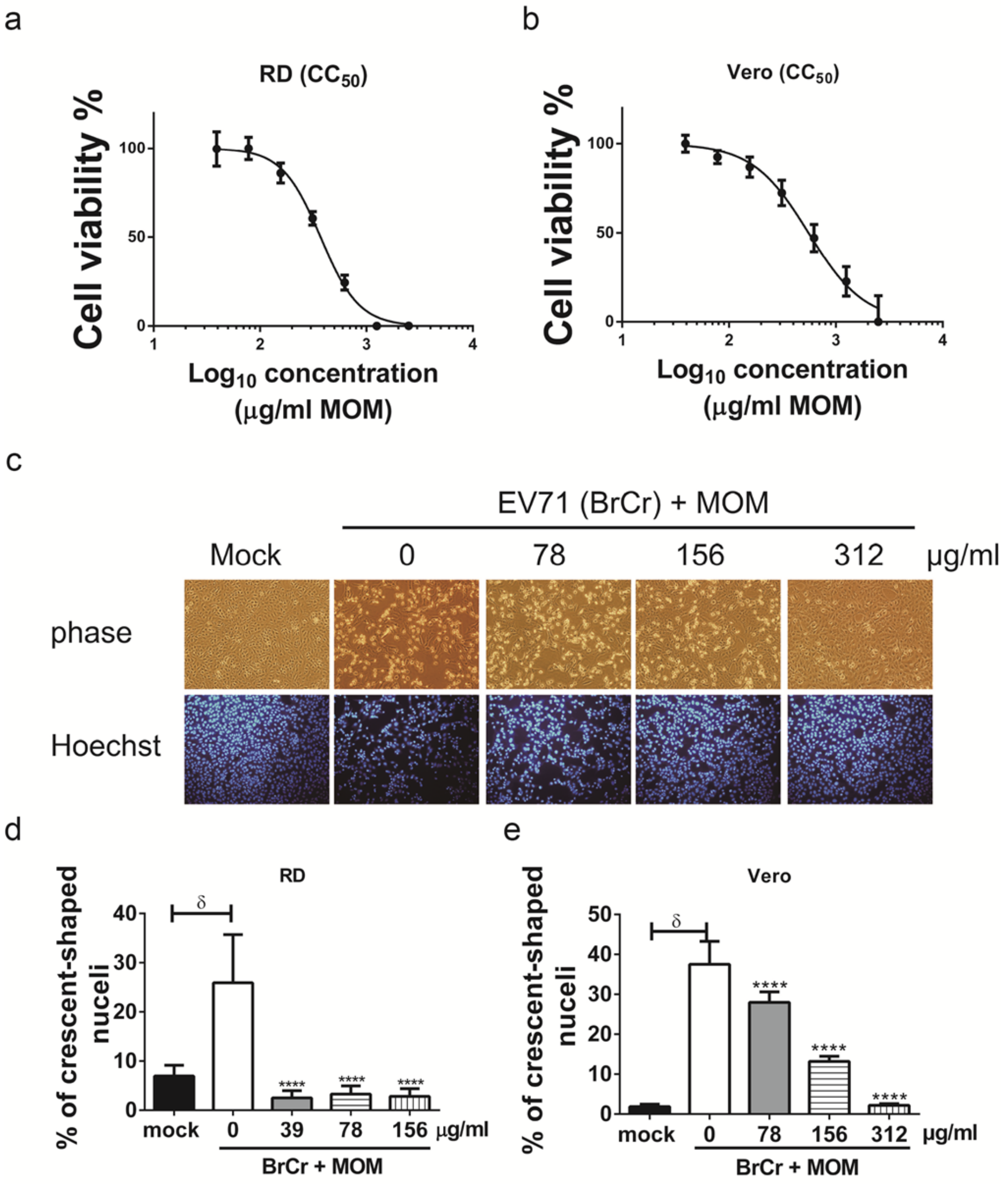

Supplementary Figure S1. The MOM causes low cytotoxicity to RD and Vero cells, and reduces CPE of EV71-infected cells. (a & b) The cytotoxicity of MOM. RD (a) and Vero (b) cells were incubated with serially diluted MOM in DMEM/2% FBS for 24 h, fixed and stained with Hoechst 33342, and the cell viability was measured with IN Cell Analyzer 1000. Data are means ± SD of three experiments. (c-e) The inhibitory effect of MOM on CPE of EV71-infected cells. (c) Vero cells were infected with BrCr at an m. o. i. of 1 in the absence or presence of 78, 156 or 312 μg/ml MOM for 16 h. The cells were then fixed with 10% formalin, stained with Hoechst 33342, and observed under fluorescence microscope. Cells displaying CPE are characterized by cells rounding, chromatin condensation and formation of crescent-shaped nuclei. A

representative experiment out of three is shown. RD (d) and Vero (e) cells were infected with BrCr at an m. o. i. of 0.05 and 1, respectively, for 1 h. Cells were treated with serially diluted MOM in DMEM/2% FBS for 24 h. They were fixed and stained with Hoechst 33342. The percentage of cells showing crescent-shaped nuclei was analyzed using IN Cell Analyzer 1000. Data are means ± SD of three experiments. δ, $P<0.0001$, vs. mock-infected cells; ****, $P< 0.0001$, vs. infected cells without treatment.

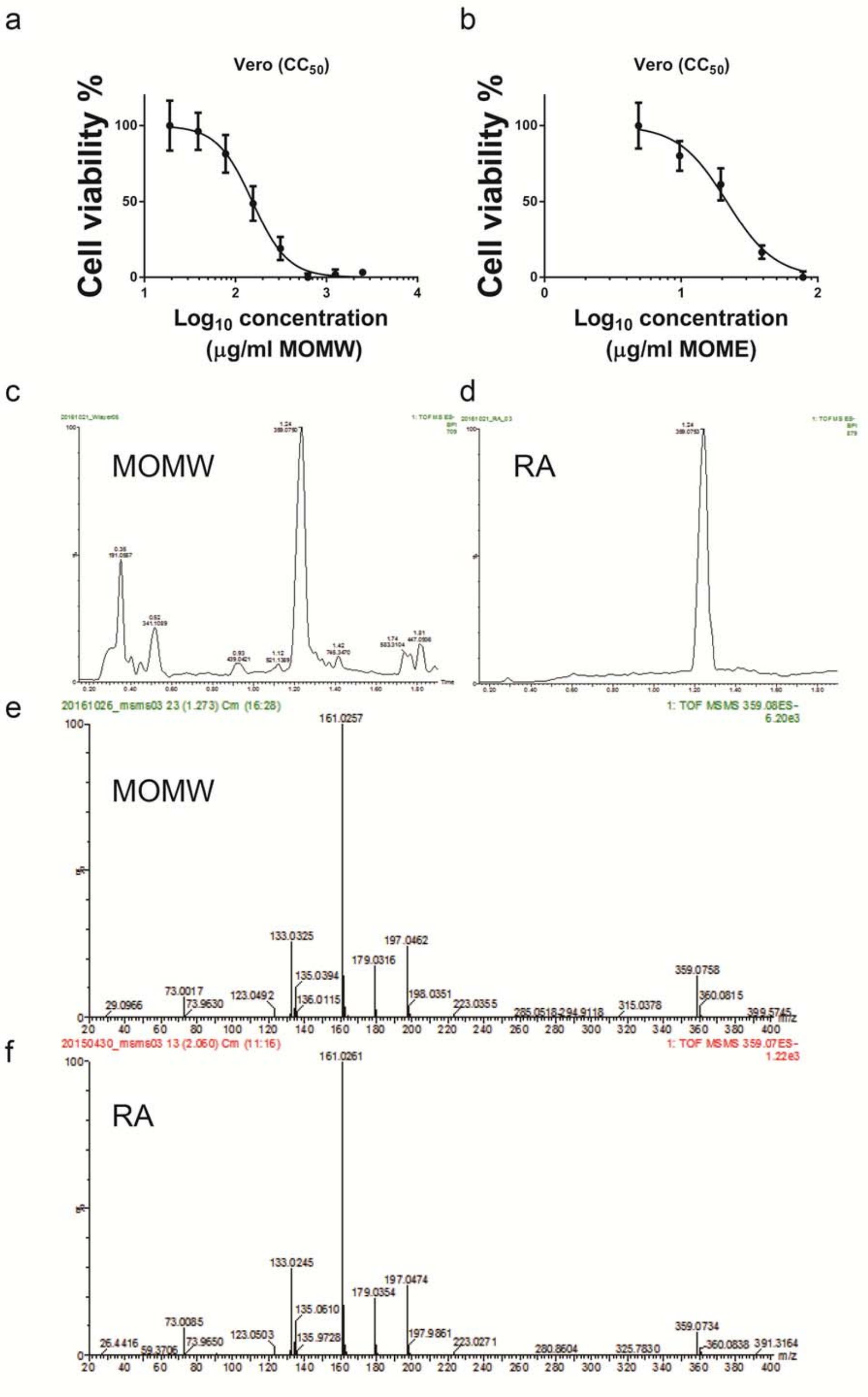

Supplementary Figure S2. (a & b) The cytotoxicity of MOMW and MOME. Vero cells were treated with serially diluted MOMW and MOME for 72 h, and cell viability was measured using neutral red assay. Data are means ± SD of three experiments. (c & d) Identification of RA as a constituent of MOM. MOMW and RA purchased from Sigma-Aldrich (St. Louis, MO, USA) were dissolved in DMSO and subject to

UPLC-MS. The base peak chromatograms of MOMW (c) and RA (d) are shown. The compound with *m/z* = 359.07 identified in Fig. 2c was subject to tandem mass spectrometry analysis in negative ion mode (with a trap collision energy of 17V). The MS/MS spectrum (e) of the compound with *m/z* = 359.07 is identical to that of RA (f).

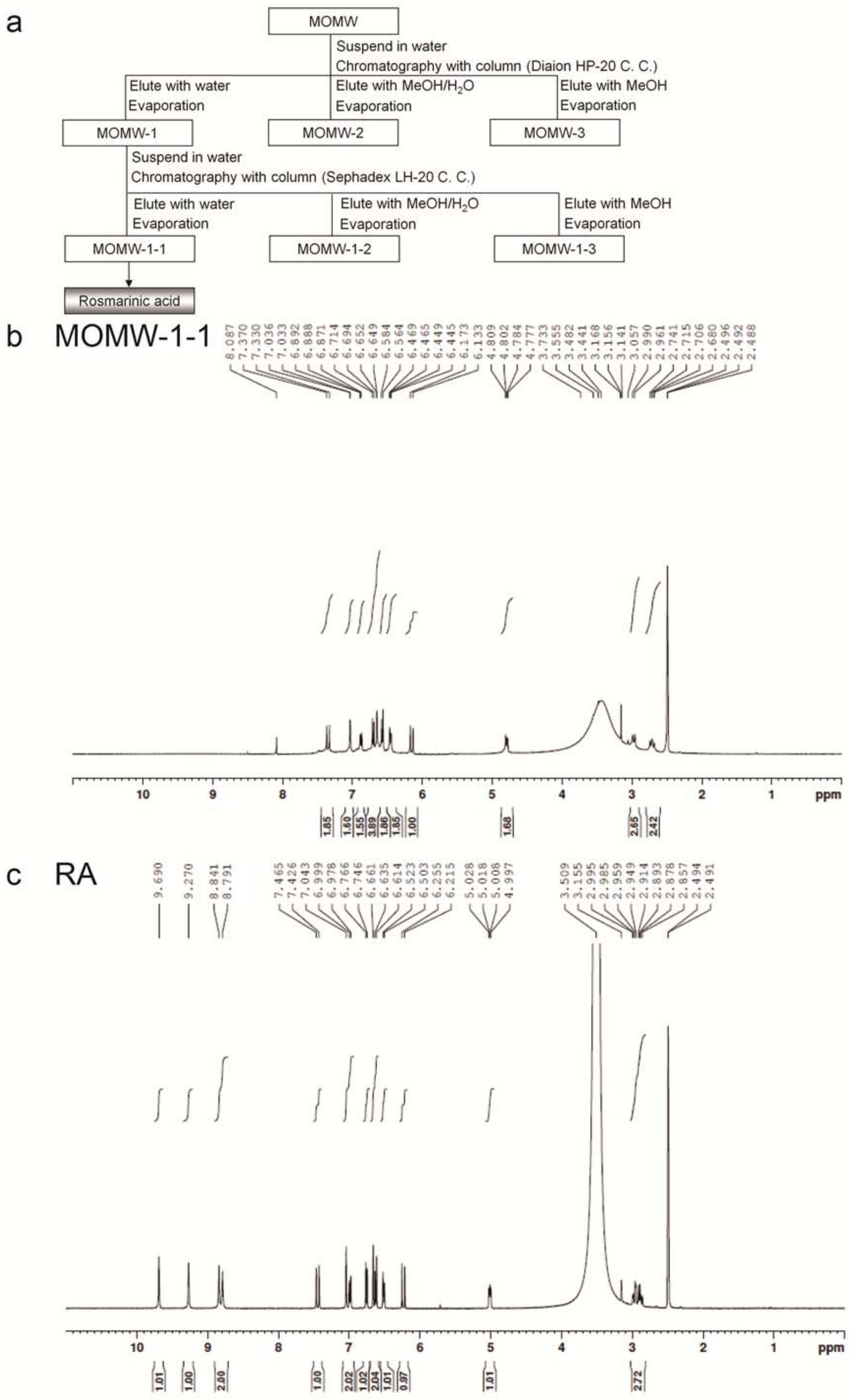

Supplementary Figure S3. Purification of MOMW and characterization by proton nuclear magnetic resonance (^{1}H-NMR). The MOMW, derived from the syrup of MOM, was purified by chromatography successively on Diaion HP-20 column and Sephadex LH-20 column. The eluents were water, water/methanol (1:1, V/V) and methanol. The sample eluted from Sephadex LH-20 column by water (MOMW-1-1)

was retained. This fraction was concentrated with rotary evaporator, dissolved in DMSO-*d6*, and subject to ^{1}H-NMR analysis using Bruker Avance III-400 NMR spectrometer equipped with a multinuclear broadband fluorine observe probe (BBFO, 5 mm). A 600 μl sample of MOMW-1-1 was transferred into an NMR tube and spectra were generated. NMR spectra were recorded at a resonance frequency of 400 MHz. Chemical shifts are presented as δ values. For spectral comparison, RA, purchased from Sigma-Aldrich (St. Louis, MO, USA), was dissolved in DMSO-*d6* and analyzed in parallel. (a) The workflow for the purification of MOMW-1-1 is shown. The NMR spectra for MOMW-1-1 (b) and RA (c) are shown.

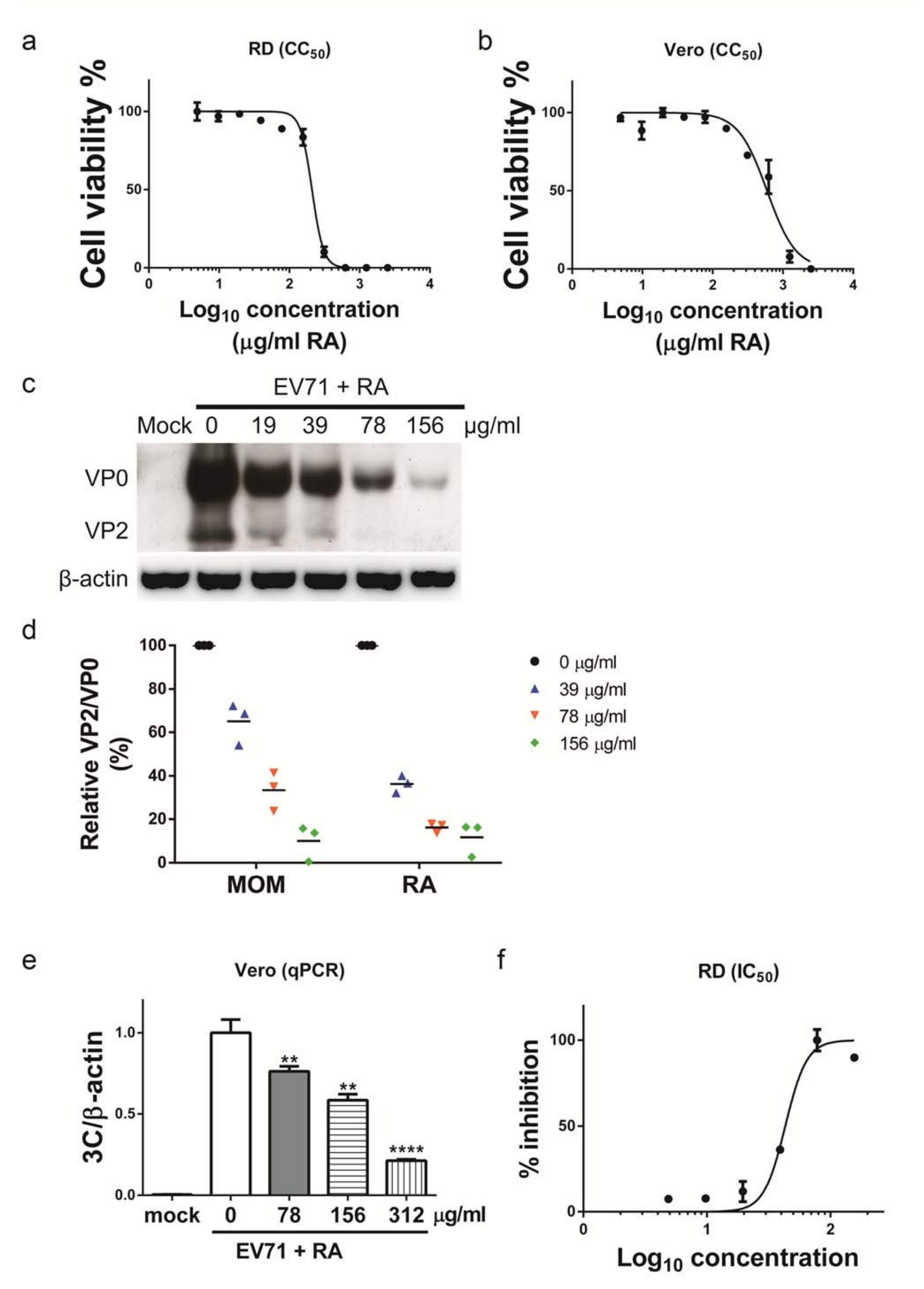

Supplementary Figure S4. RA represses EV71 infection. (a & b) The cytotoxicity of RA. RD or Vero cells were incubated with serially diluted RA for 24 h, fixed, and stained with Hoechst 33342. Cell viability was determined using IN Cell Analyzer 1000. Data are means ± SD of three experiments. (c) RD cells were infected with BrCr at an m. o. i. of 0.05 and treated with 19, 39, 78, 156 μg/ml RA for 16 h. Cell

lysates were harvested and subject to immunoblotting with antibodies against VP2 and β-actin. A representative experiment out of three is shown. (d) The blots were scanned, and band intensities were quantified using image J. The ratio of the level of VP2 to that of VP0 (VP2/VP0) of MOM- (Fig. 1b) or RA-treated (Supplementary Figure S4c) cells is expressed as the percentage of the VP2/VP0 ratio of the untreated infected cells. (e) Vero cells were mock- or infected with BrCr at an m. o. i. of 1 in absence or presence of 78, 156 or 312 μg/ml RA for 16 h. Total RNA was isolated. The level of EV71 genomic copy was determined by quantitative reverse transcription PCR, and normalized to the level of β-actin. Data are expressed relative to that of untreated cells. The results are means ± SD of three separate experiments. **, $P<0.01$, ****, $P<0.0001$, vs. infected cells without treatment. (f) The half maximal inhibitory concentration (IC_{50}) of RA for inhibitory effect on BrCr was measured in RD cells. RD cells were infected BrCr at an m. o. i. of 10 and treated with 9.75, 19.5, 39, 78, or 156 μl/ml RA for 24 h. The cell number was measured by neutral red assay. The results are mean ± SD in triplicate.

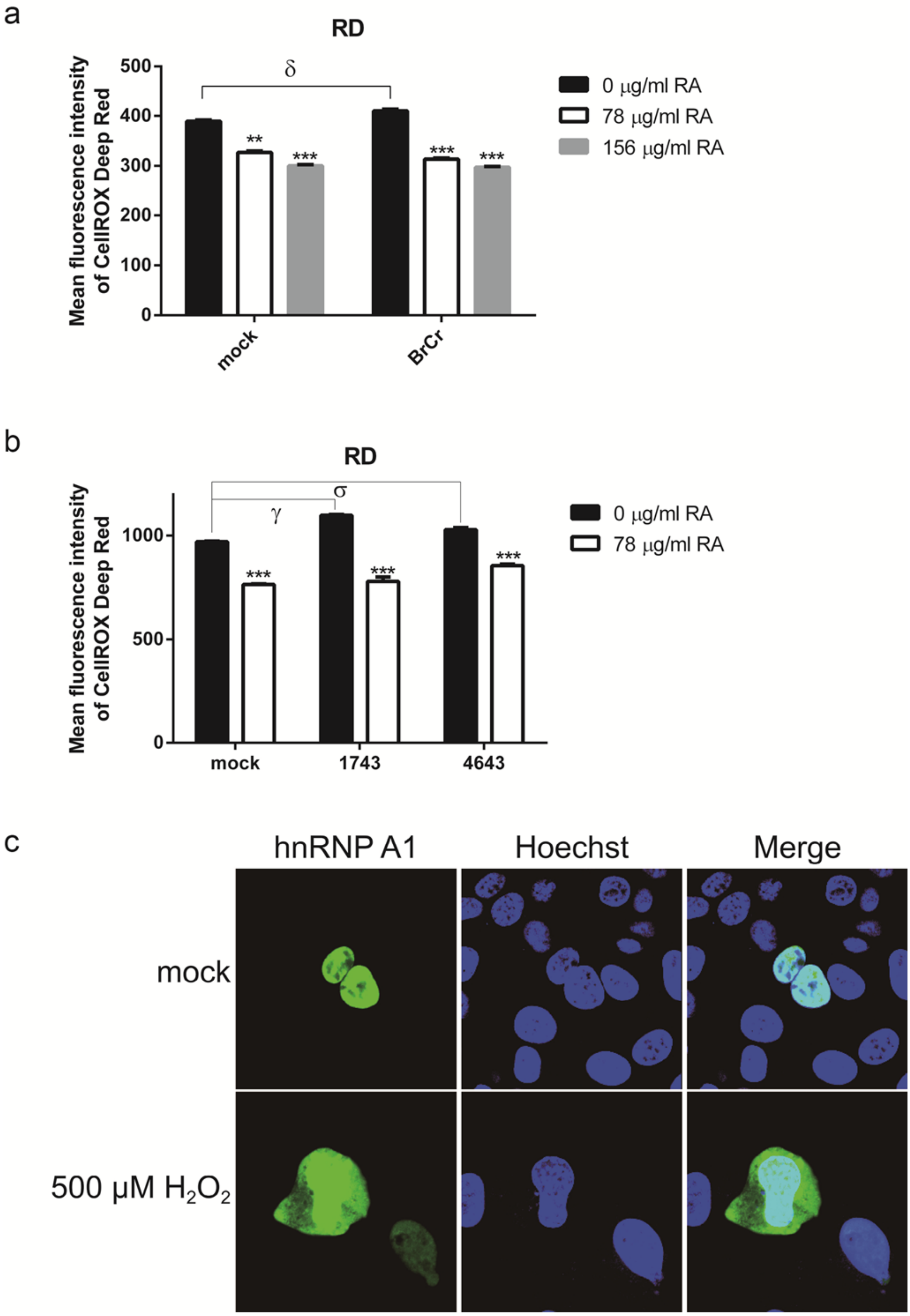

Supplementary Figure S5. RA inhibits EV71-induced generation of ROS, which activates the translocation of hnRNP A1 from nucleus to cytoplasm. (a & b) RD cells were infected with BrCr, 1743, or 4643 strains at an m. o. i. of 0.05 for 1 h, and subsequently treated with indicated concentration of RA for 24 h. Cells were stained

with CellROX Deep Red for 30 min and analyzed using flow cytometry. Data are mean ± SD of three separate experiments. **, $P<0.01$, ***, $P<0.001$, vs. cells without RA treatment. δ, $P<0.05$; BrCr-infected vs. mock-infected cells; γ, $P<0.001$; 1743-infected vs. mock-infected cells; σ, $P<0.01$; 4643-infected vs. mock-infected cells. (c) Hydrogen peroxide induced the cytoplasmic accumulation of hnRNP A1. RD cells were transfected with expression vector encoding GFP-tagged hnRNP A1 for 48 h. The transfected cells were treated without or with 500 μM H_2O_2 for 7 h, fixed, and stained with Hoechst 33342. The image was captured using confocal microscopy. A representative experiment out of three is shown.

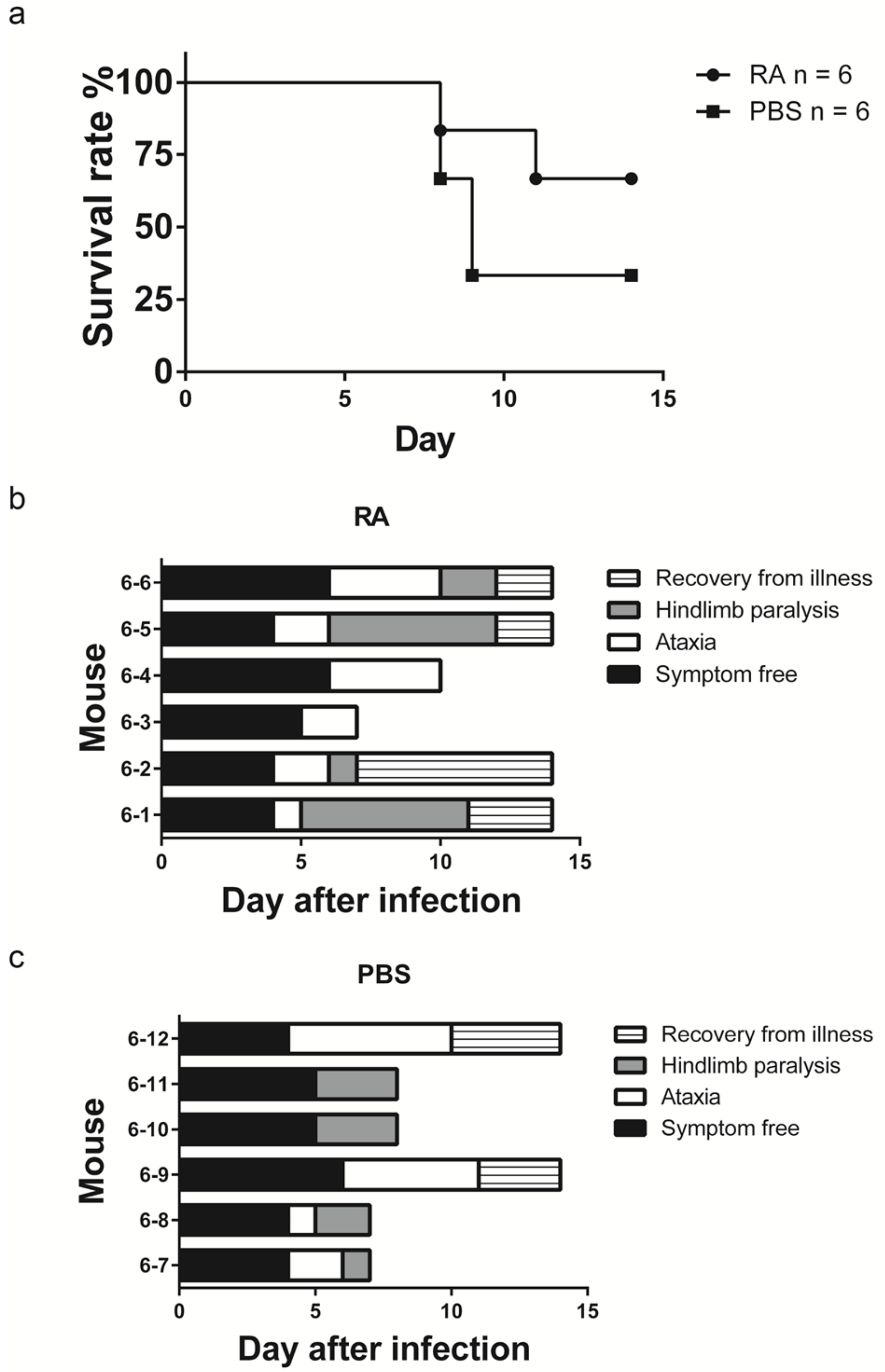

Supplementary Figure S6. RA alleviates the clinical symptoms and increases the survival rate in EV71 infected mice. (a) Seven-day-old ICR mice were intraperitoneally infected with 10^6 PFU MP4. After 1 day, EV71-infected mice were treated with single dose of RA (50 mg/kg) (n=6) or PBS (10 ml/kg) (n=6) daily for 14

days. The survival rate (a) and clinical symptoms in RA-treated (b) and PBS-treated mice (c) were monitored, and recorded daily for 14 days. The clinical symptoms, including ataxia, hind limb paralysis and death, were recorded.

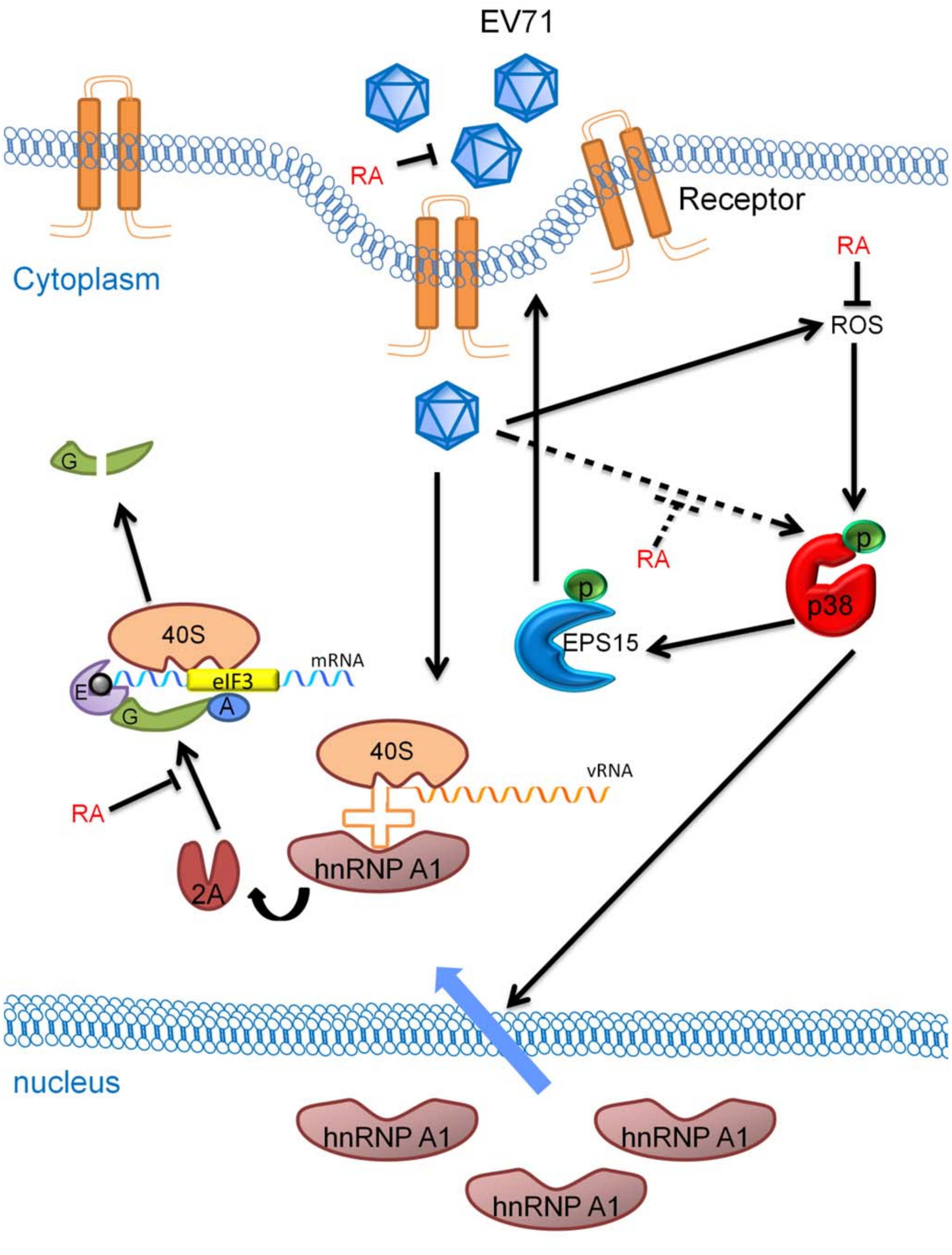

Supplementary Figure S7. A proposed model for antiviral action of RA. RA suppresses multiple pathways in EV71-infected cells. RA interacts with viral particles to interfere with their attachment to receptor. It suppresses the protease $2A^{pro}$-mediated hydrolysis of eIF4G, which acts as a scaffold of eIF4F and interacts with eIF4E (cap-binding protein, labeled E) and eIF4A (helicase, labeled A). Besides,

RA scavenges ROS, and inhibits ROS-mediated activation of p38 kinase. This leads to diminished cytoplasmic accumulation of hnRNP A1 and EPS15 phosphorylation. hnRNP A1 binds to IRES of EV71, and facilitates the recruitment of 40S ribosome for initiation of viral translation. EPS15 is known to modulate the clathrin-mediated endocytosis and membrane trafficking. An arrow-ending line indicates a route or pathway; a bar-ending line indicates an inhibitory effect; dash line shows an effect that cannot be excluded in the present model.

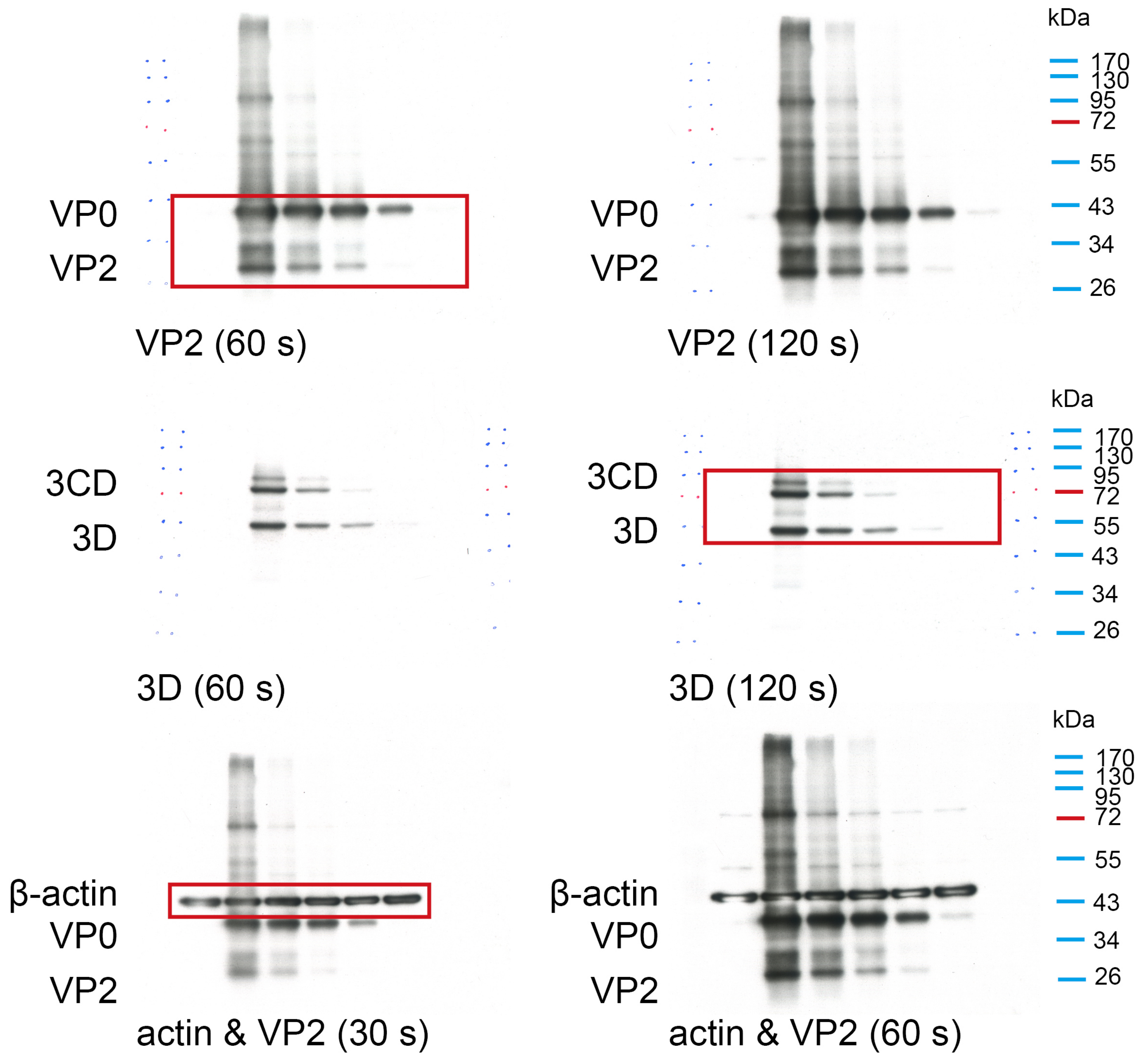

Supplementary Figure S8. Uncropped immunoblot image for Figure 1b. Red box marks the borders of the cropped image for the indicated protein. Numbers on the right of image indicate the positions of molecular weight markers (kDa). Images generated by exposure of the immunoblot for the indicated periods are shown.

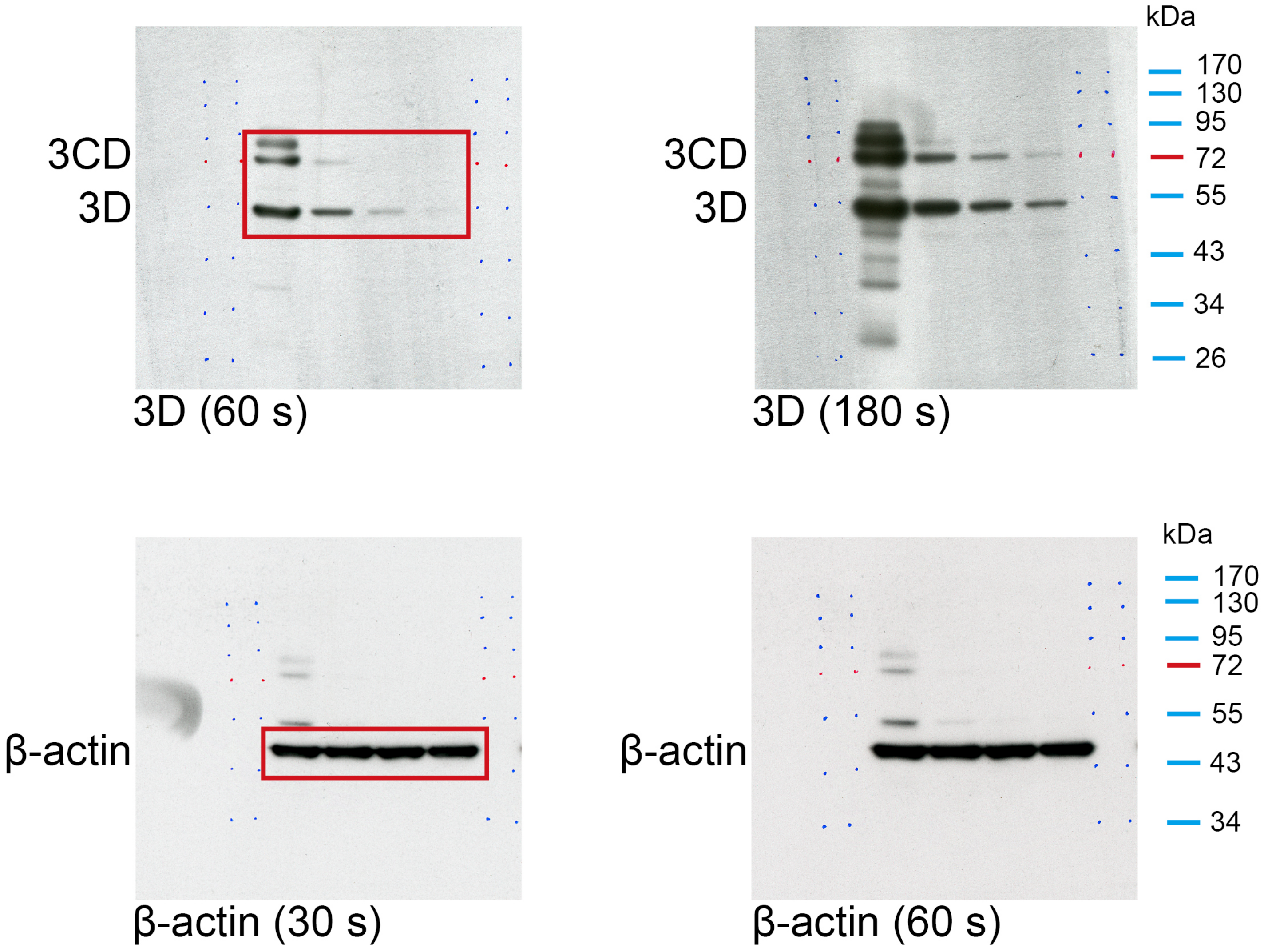

Supplementary Figure S9. Uncropped immunoblot image for Figure 3b. Red box marks the borders of the cropped image for the indicated protein. Numbers on the right of image indicate the positions of molecular weight markers (kDa). Images generated by exposure of the immunoblot for the indicated periods are shown.

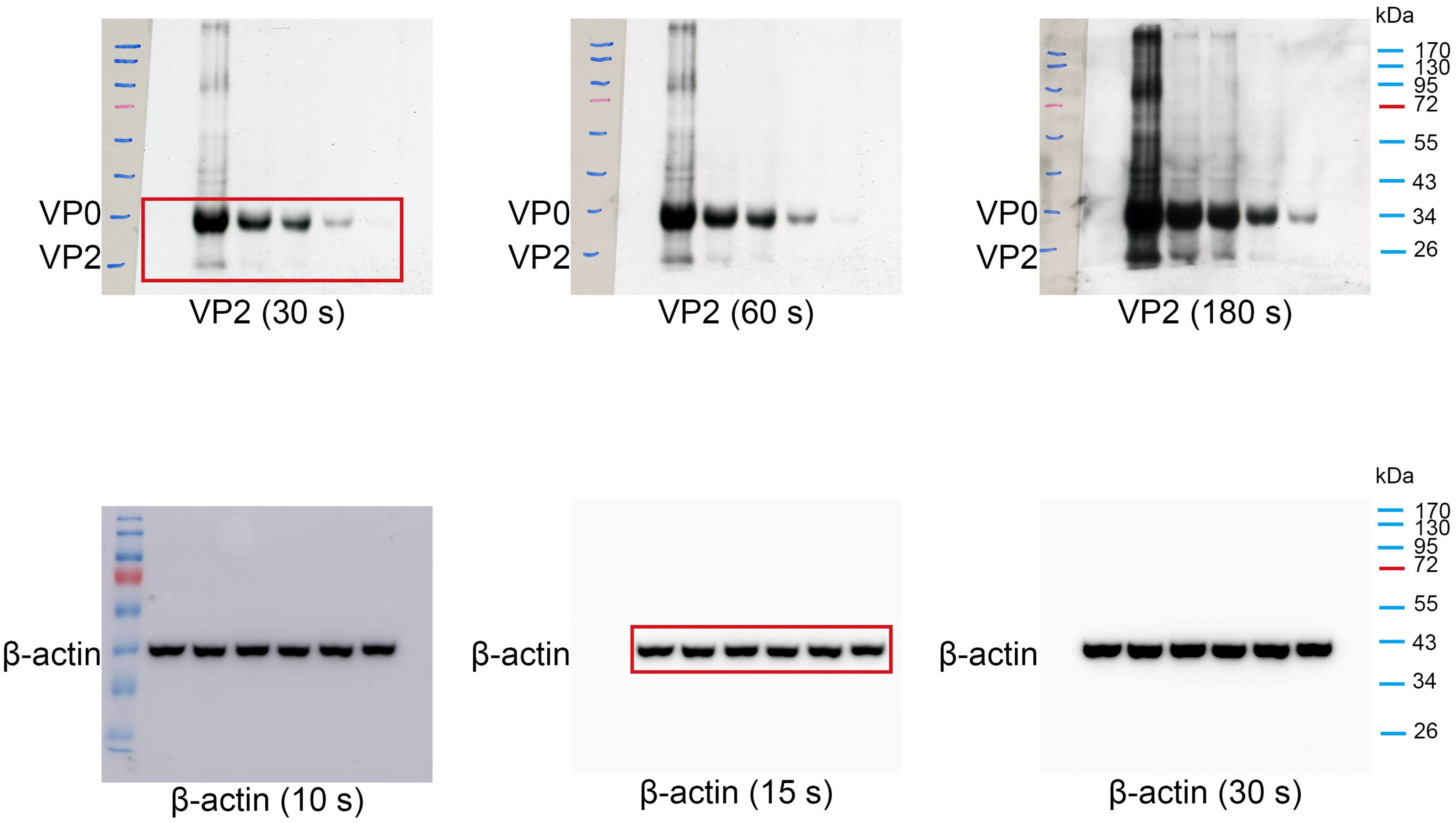

Supplementary Figure S10. Uncropped immunoblot image for Figure 3c. Red box marks the borders of the cropped image for the indicated protein. Numbers on the right of image indicate the positions of molecular weight markers (kDa). Images generated by exposure of the immunoblot for the indicated periods are shown.

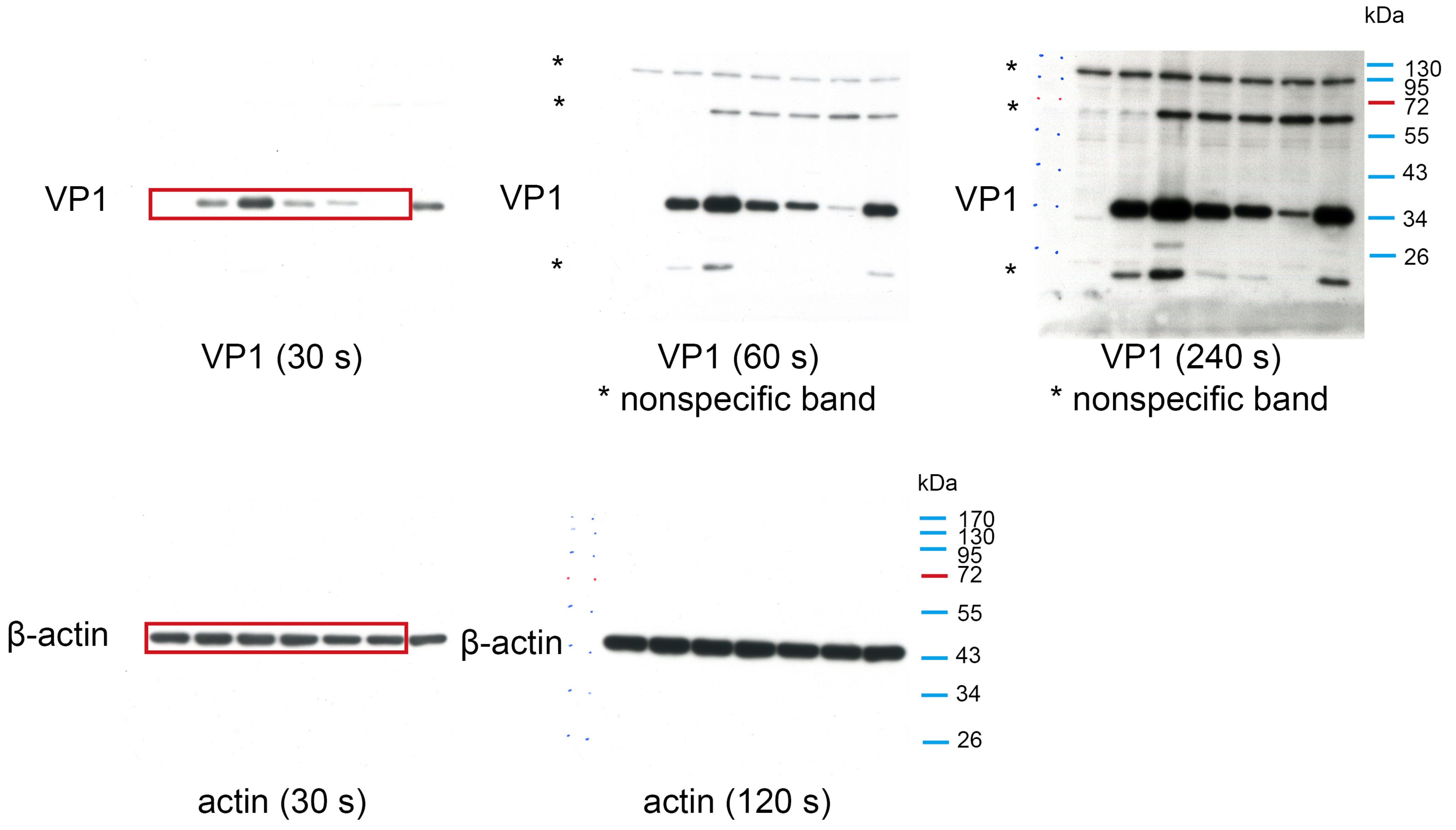

Supplementary Figure S11. Uncropped immunoblot image for Figure 4b. Red box marks the borders of the cropped image for the indicated protein. Numbers on the right of image indicate the positions of molecular weight markers (kDa). Images generated by exposure of the immunoblot for the indicated periods are shown.

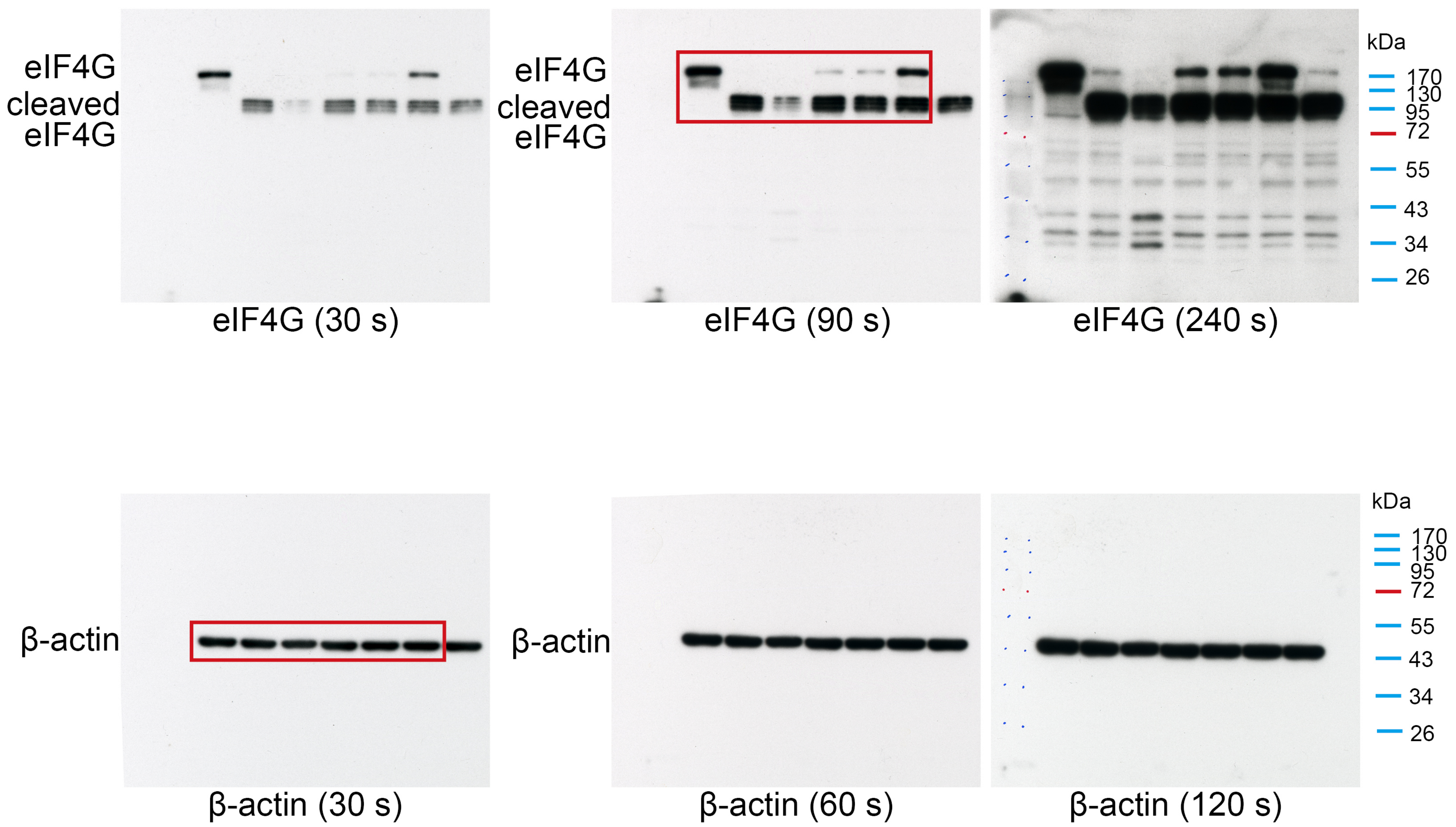

Supplementary Figure S12. Uncropped immunoblot image for Figure 5c. Red box marks the borders of the cropped image for the indicated protein. Numbers on the right of image indicate the positions of molecular weight markers (kDa). Images generated by exposure of the immunoblot for the indicated periods are shown.

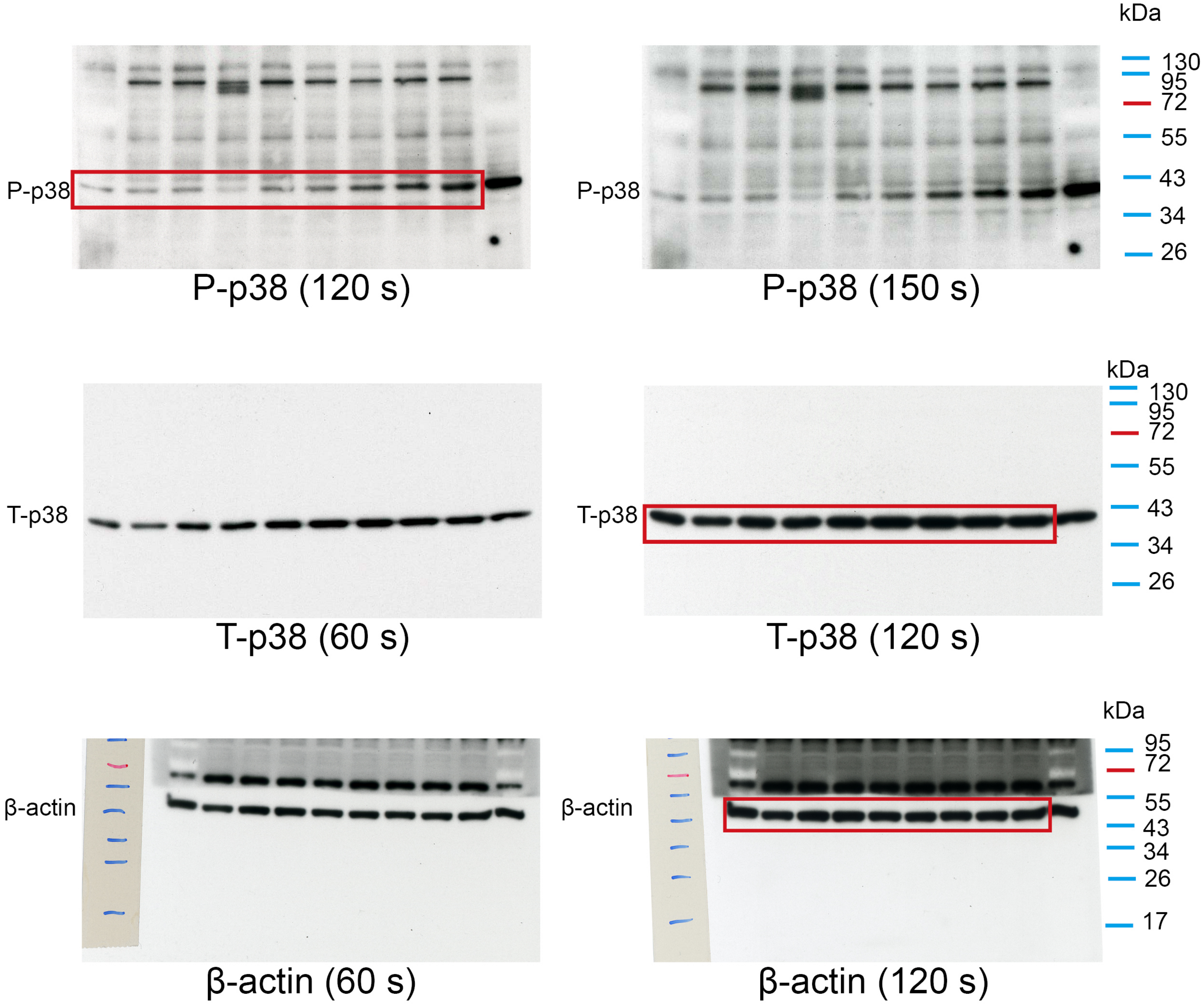

Supplementary Figure S13. Uncropped immunoblot image for Figure 6a. Red box marks the borders of the cropped image for the indicated protein. Numbers on the right of image indicate the positions of molecular weight markers (kDa). Images generated by exposure of the immunoblot for the indicated periods are shown.

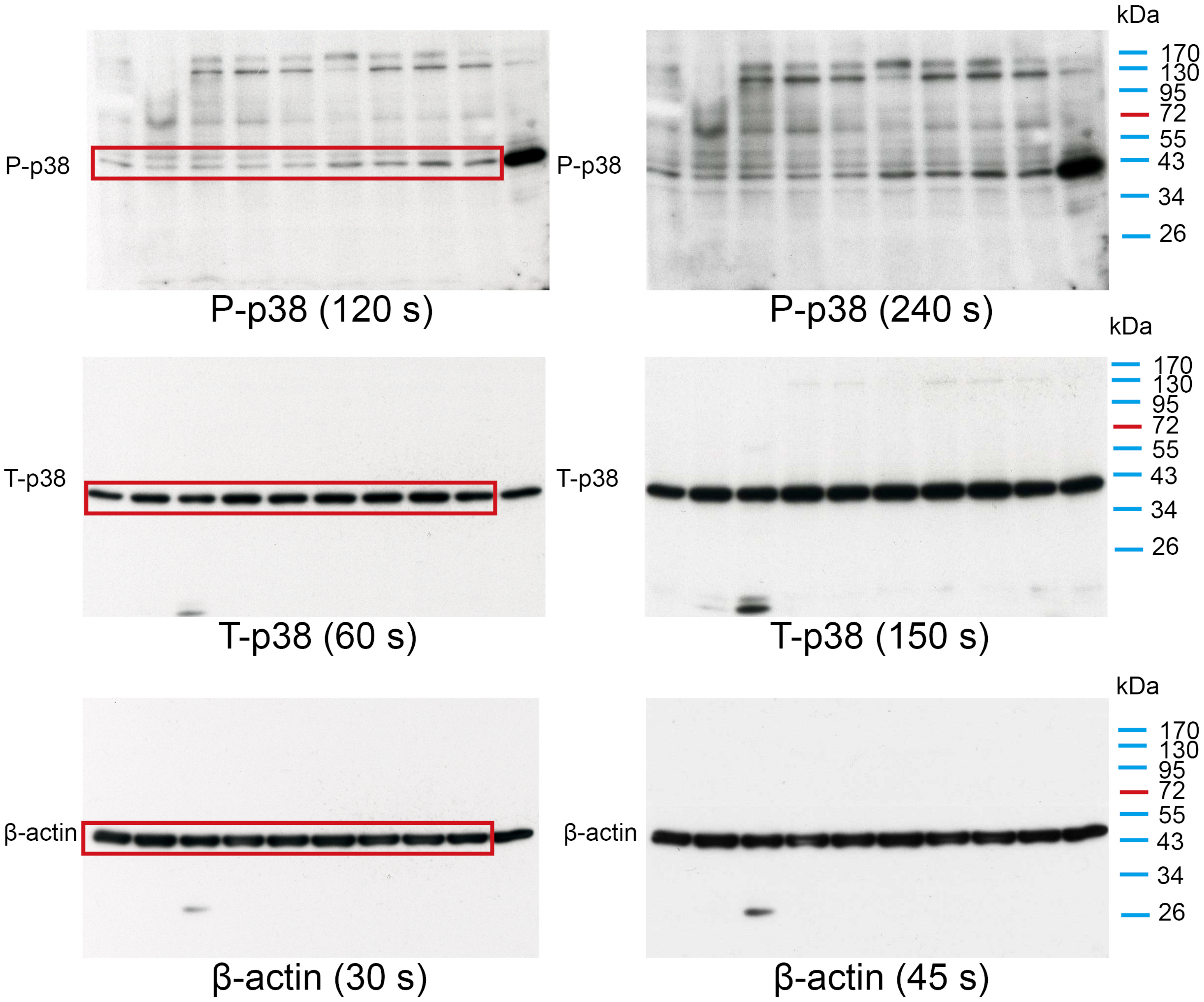

Supplementary Figure S14. Uncropped immunoblot image for Figure 6b. Red box marks the borders of the cropped image for the indicated protein. Numbers on the right of image indicate the positions of molecular weight markers (kDa). Images generated by exposure of the immunoblot for the indicated periods are shown.

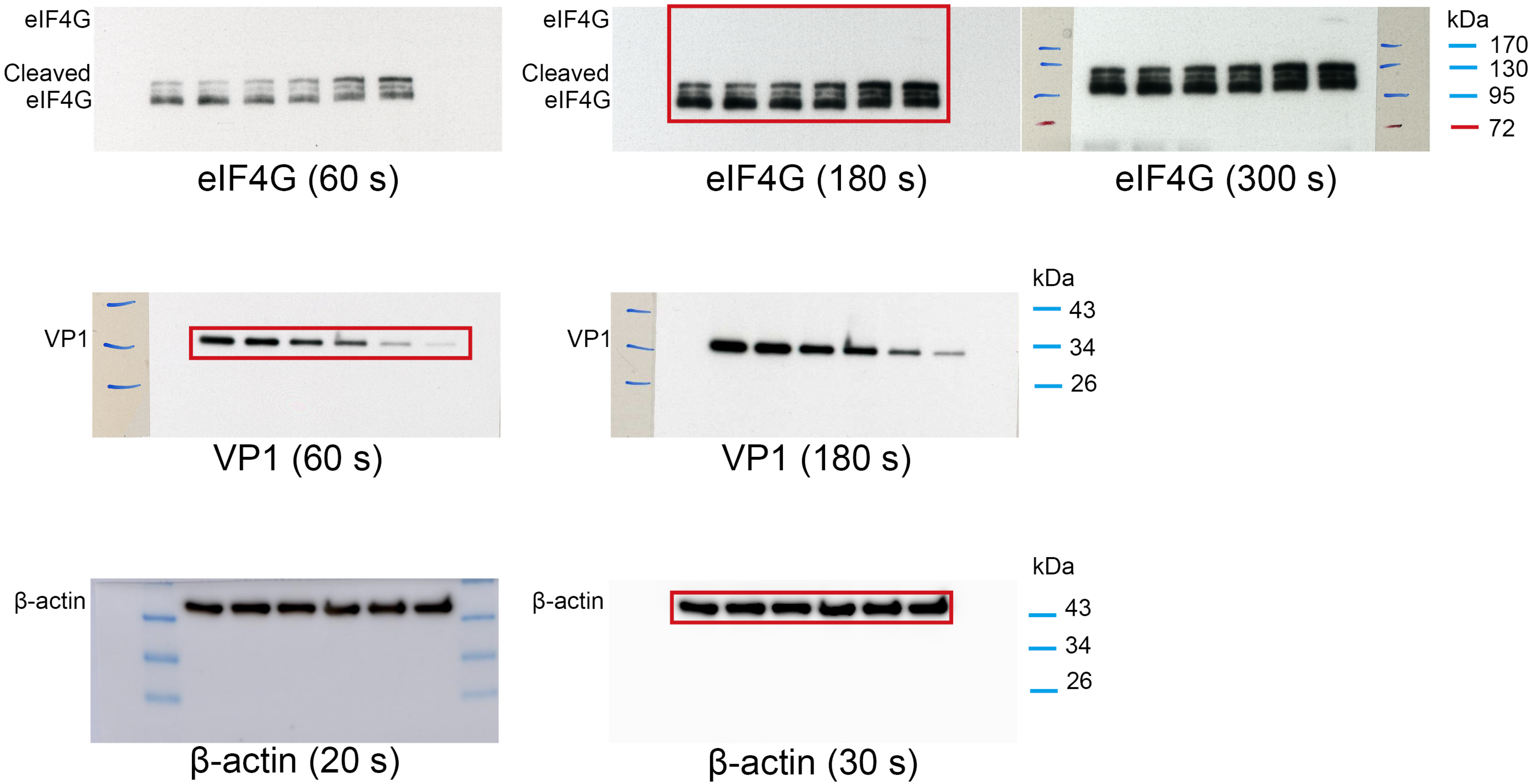

Supplementary Figure S15. Uncropped immunoblot image for Figure 6c. Red box marks the borders of the cropped image for the indicated protein. Numbers on the right of image indicate the positions of molecular weight markers (kDa). Images generated by exposure of the immunoblot for the indicated periods are shown.

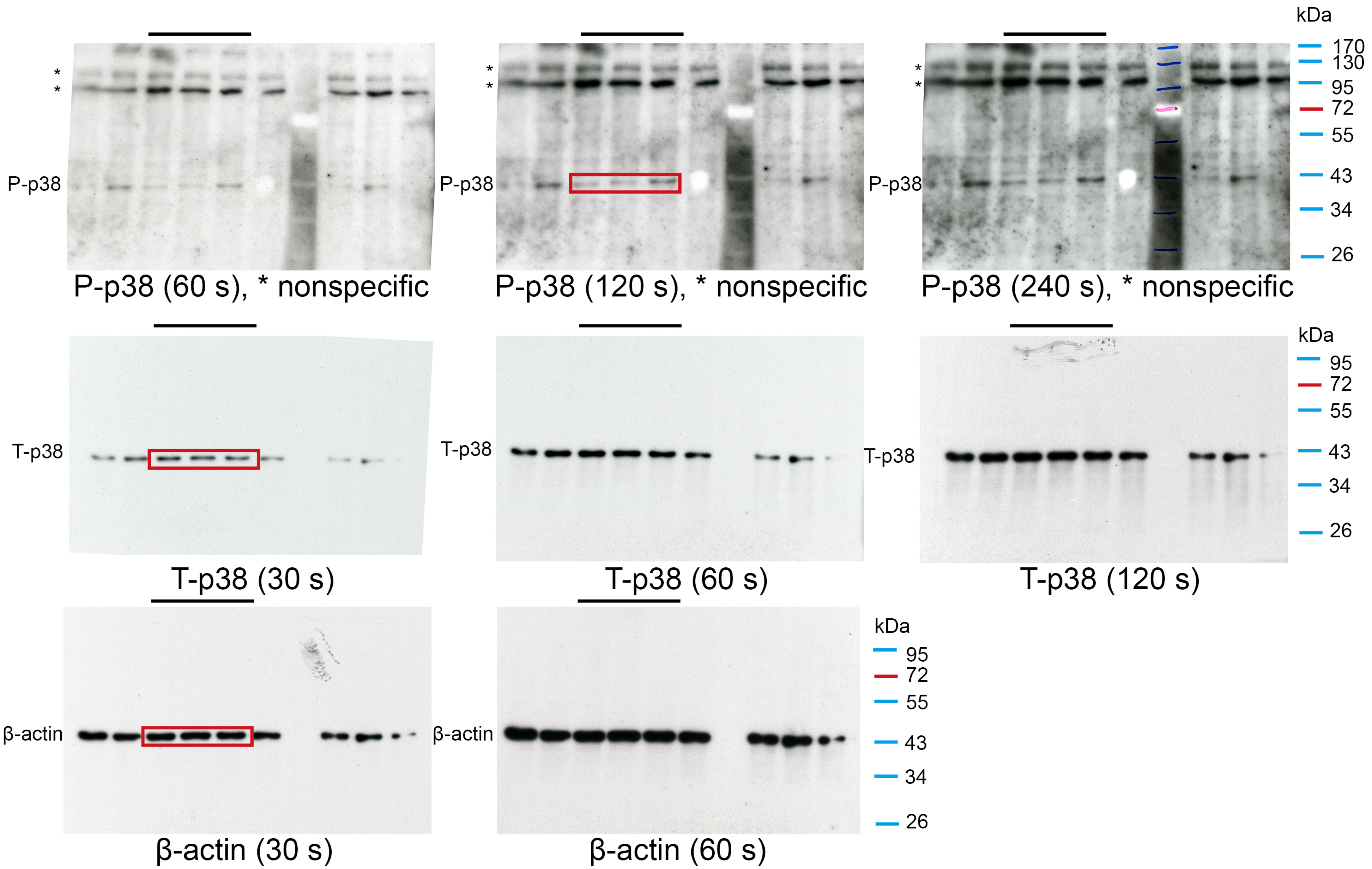

Supplementary Figure S16. Uncropped immunoblot image for Figure 7b. Red box marks the borders of the cropped image for the indicated protein. Numbers on the right of image indicate the positions of molecular weight markers (kDa). Images generated by exposure of the immunoblot for the indicated periods are shown.

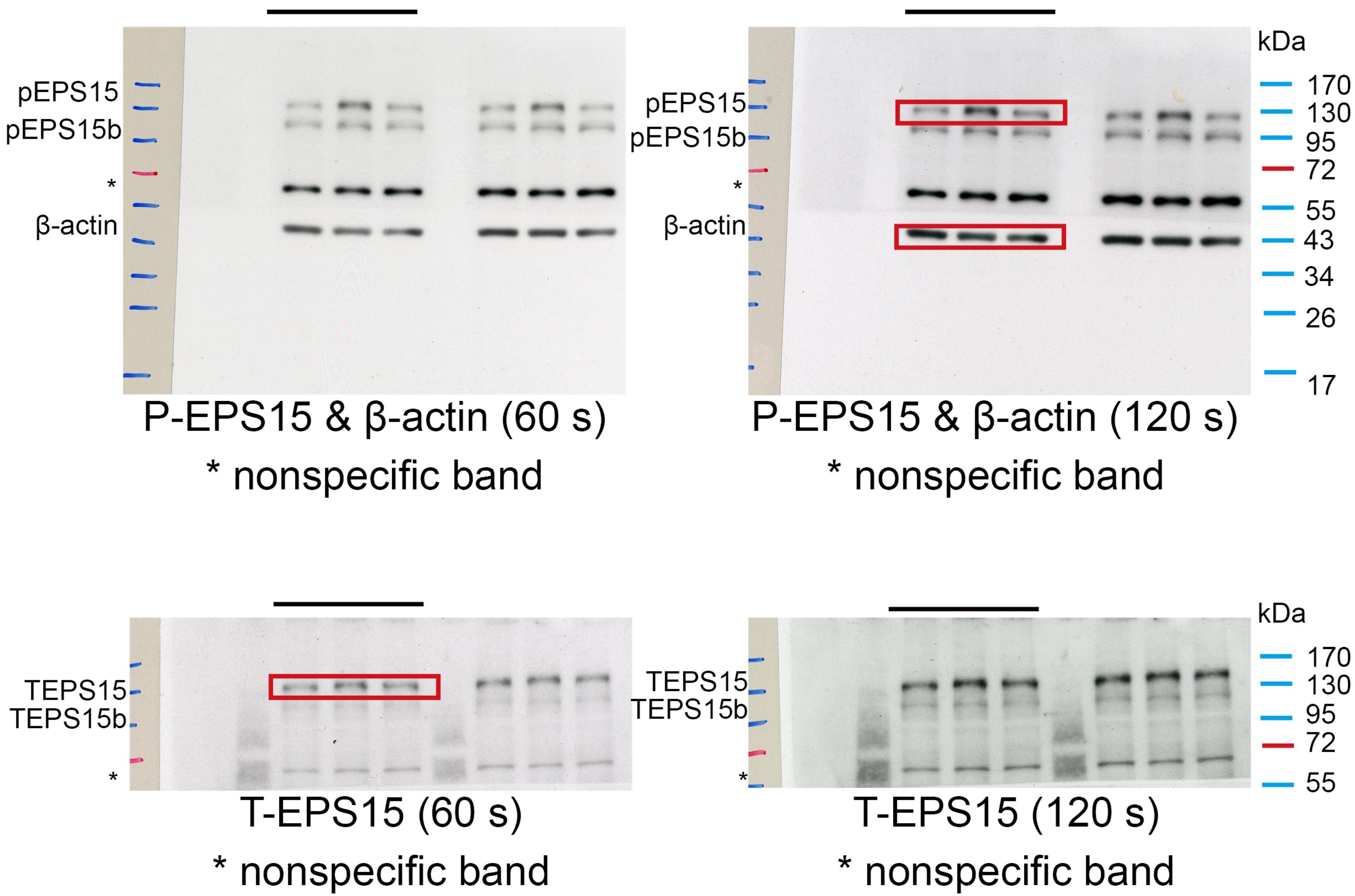

Supplementary Figure S17. Uncropped immunoblot image for Figure 8a. Red box marks the borders of the cropped image for the indicated protein. Numbers on the right of image indicate the positions of molecular weight markers (kDa). Images generated by exposure of the immunoblot for the indicated periods are shown.

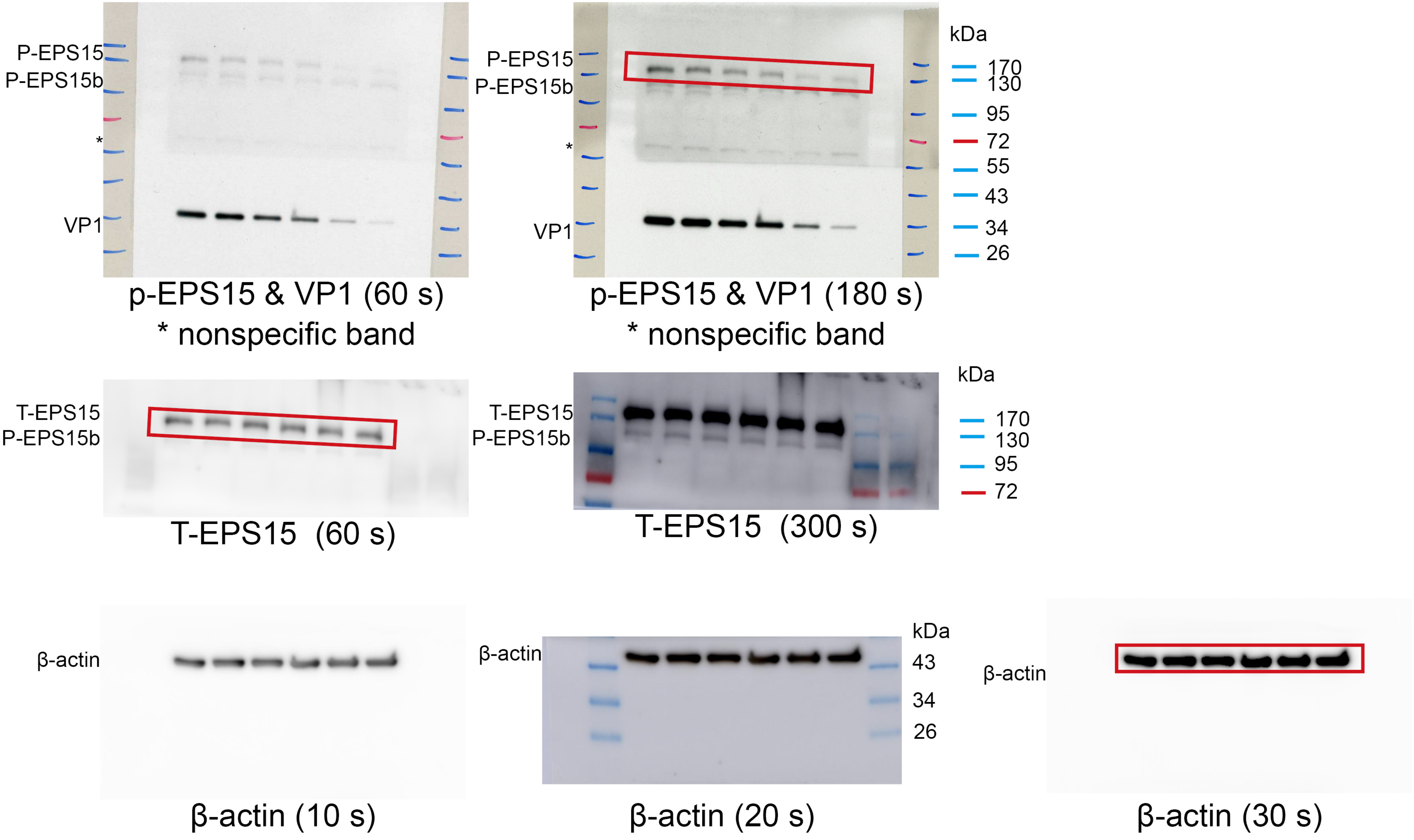

Supplementary Figure S18. Uncropped immunoblot image for Figure 8b. Red box marks the borders of the cropped image for the indicated protein. Numbers on the right of image indicate the positions of molecular weight markers (kDa). Images generated by exposure of the immunoblot for the indicated periods are shown.

Supplementary Methods

Cytopathic effect assay

Monolayer of RD cells (5 x 10^4 per well) or Vero cells (2 x 10^5 per well) were cultured in 12-well plates and mock- or infected with EV71 at an m. o. i. of 0.05 for RD cells and 1 for Vero cells, respectively. After 1 hour of viral absorption, an equal volume of medium containing 2% FBS and an indicated concentration of extract or RA was added to the wells. Cells were incubated at 37 ^{o}C in a humidified environment of 5% CO_2 for 16 h. The treated cells were subsequently fixed in 10% formalin for 30 min, and stained with Hoechst 33342 (5 μg/ml) for 30 min. Morphology of cells and nuclei was observed under fluorescence microscopy (Olympus, Japan). Quantitative data on CPE was obtained by high throughput imaging analysis with IN Cell Analyzer 1000 (GE Healthcare Life Sciences, Pittsburgh, PA, USA).

IC_{50} determination

For determination of IC_{50} of MOM (or RA), RD cells were infected with BrCr at an m. o. i. of 10 in the presence of 9.75, 19.5, 39, 78, or 156 μg/ml of MOM (or 9.75, 19.5, 39, 78, or 156 μl/ml RA) for 24 h. The cell number was determined by neutral red assay (with reference to a standard curve). The % inhibition was calculated as $\frac{N_{SI}-N_I}{N_{mock}-N_I}\times 100\%$, where N_{SI}, N_I, N_{mock} are the number of infected cells treated with indicated concentration of RA, number of untreated infected cells, and number of untreated control cells, respectively.

LC-MS method.

LC-MS analysis was carried out Waters Acquity ultra performance liquid chromatography (UPLC) system coupled to a quadrupole time-of-flight (Q-TOF) mass spectrometer (Waters, Milford, MA, USA). Chromatographic separation was performed on an Acquity BEH C18 column (Waters) at a flow rate of 0.5 ml/min. Column temperature was maintained at 45 °C. Solvent A consisted of water and solvent B of acetonitrile. Solvent gradient was applied as follows: 0–2.5 min, 1–48% solvent B; 2.5–3 min, 48-98% solvent B; 3–4.2 min, 98% solvent B; 4.2–6 min, 1% solvent B for re-equilibration. Data were acquired by Synapt HDMS system in negative-ion ESI mode. The capillary and cone voltage were set at 2000 V and 35 V, respectively. Desolvation gas flow rate was set at 700 L per hour, and gas flow was maintained at 25 L per hour. The desolvation and source temperatures were set at 300 °C and 80 °C, respectively. MS data were acquired over a range of *m/z* of 20 to 990 at

a rate of 0.1 s per scan. Data were collected in centroid mode. Sulfadimethoxine was used as a reference compound (*m/z* of 309.0658 in ESI-mode). For MS/MS analysis, trap collision energy was set at 17 V to induce dissociation of mass-selected parent ions.

Animal study

The CD1 (ICR) mice, acquired from BioLASCO Taiwan Co., Ltd (Taipei, Taiwan), were maintained in the laboratory animal center of Chang Gung Memorial Hospital at Linkou, Taiwan. The mice were fed the standard laboratory chow diet. Seven-day-old ICR mice were intraperitoneally injected with 2 x 10^6 PFU MP4 in 10 μl[1]. One day after infection, infected mice were intraperitoneally injected with phosphate buffered saline or RA (50 mg/kg) daily for 14 days. They were monitored daily for 14 days for body weight change, clinical symptoms and survival.

Ethic statement

All animal methods and care described in the present study were carried out in accordance with national guide. They were approved by the Institutional Animal Care and Use Committee of Chang Gung Memorial Hospital at Linkou (IACUC No. 2012101801).

References

1. Wang, Y. F. *et al.* A mouse-adapted enterovirus 71 strain causes neurological disease in mice after oral infection. *J. Virol.* 78, 7916-7924, doi:10.1128/JVI.78.15.7916-7924.2004 (2004).
